# MYC/TET3‐Regulated TMEM65 Activates OXPHOS‐SERPINB3 Pathway to Promote Progression and Cisplatin Resistance in Triple‐Negative Breast Cancer

**DOI:** 10.1002/advs.202500421

**Published:** 2025-06-23

**Authors:** Yin‐Ling Zhang, Min‐Ying Huang, Shao‐Ying Yang, Jia‐Yang Cai, Qian Zhao, Fang‐Lin Zhang, Xin Hu, Zhi‐Min Shao, Li Liao, A‐Yong Cao, Da‐Qiang Li

**Affiliations:** ^1^ Fudan University Shanghai Cancer Center and Institutes of Biomedical Sciences Fudan University Shanghai 200032 China; ^2^ Cancer Institute Shanghai Medical College Fudan University Shanghai 200032 China; ^3^ Department of Breast Surgery Fudan University Shanghai Cancer Center Shanghai 200032 China; ^4^ Shanghai Key Laboratory of Breast Cancer Shanghai Medical College Fudan University Shanghai 200032 China; ^5^ Precision Cancer Medicine Center Fudan University Shanghai Cancer Center Shanghai 200032 China; ^6^ Department of Oncology The Second Affiliated Hospital Guangzhou Medical University Guangzhou Guangdong 510260 China

**Keywords:** cancer stemness, chemoresistance, mitochondrial metabolism, transmembrane protein, triple‐negative breast cancer

## Abstract

Triple‐negative breast cancer (TNBC) is the most lethal subtype of breast cancer due to its aggressive clinical features and the lack of effective targeted therapeutics. Mitochondrial metabolism is intimately linked to TNBC progression and therapeutic resistance and is an attractive therapeutic target for TNBC. Here, it is first reported that human transmembrane protein 65 (TMEM65), a poorly characterized mitochondrial inner‐membrane protein‐encoding gene in human cancer, acts as a novel oncogene in TNBC to promote tumor growth, metastasis, and cisplatin resistance both in vivo and in vitro. Transcription factor MYC and DNA demethylase ten‐eleven translocation 3 (TET3) coordinately upregulate TMEM65 in TNBC, and its upregulation is associated with poor patient survival. Moreover, pharmacological inhibition or knockdown of MYC and TET3 attenuates TMEM65‐driven TNBC progression. Mechanistic investigations reveal that TMEM65 enhances mitochondrial oxidative phosphorylation and its byproduct reactive oxygen species (ROS) production. Increased ROS induces the expression of hypoxia‐inducible factor 1α (HIF1α), which in turn transcriptionally activates serpin family B member 3 (SERPINB3) to enhance TNBC stemness, thus leading to TNBC progression and cisplatin resistance. Collectively, these findings identify TMEM65 as a vital oncogene of TNBC, unveil its regulatory mechanisms, and shed light on its potential role in TNBC therapy.

## Graphical abstract

Triple‐negative breast cancer (TNBC) remains a major challenge in breast cancer management as it lacks effective therapeutic targets. This study uncovers that MYC/TET3‐regulated mitochondrial inner‐membrane protein TMEM65 promotes TNBC progression and cisplatin resistance by activating the OXPHOS‐SERPINB3 pathway and reveals TMEM65 as a potential therapeutic target for TNBC.

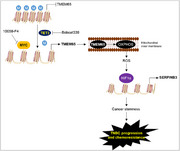

## 1 Introduction

Triple‐negative breast cancer (TNBC) is a specific subtype of breast cancer, which lacks the expression of estrogen and progesterone receptors and human epidermal growth factor receptor 2 (HER2) and accounts for about 15% of all breast cancer cases.^[^ ^1^ ^]^ Clinically, TNBC exhibits aggressively clinicopathological features with a high incidence of early recurrence and distant metastases and the worst outcomes.^[^ ^1^ ^]^ Due to the absence of validated therapeutic targets, chemotherapy is the mainstay of systemic treatment for patients with both the early and advanced stages of TNBC.^[^ ^2^ ^]^ In this context, platinum‐based regimens (e.g., cisplatin) are widely used in the neoadjuvant and adjuvant therapy of TNBC.^[^ ^3^, ^4^ ^]^ However, many patients are resistant to these drugs and quickly relapse.^[^ ^4^, ^5^ ^]^ Therefore, there is an urgent need to elucidate the molecular mechanisms of TNBC progression and chemoresistance and to identify novel targets for TNBC therapy.

Accumulating evidence shows that cancer stem cells (CSCs) are potential causes of recurrence, metastasis, and chemoresistance in TNBC.^[^ ^6^, ^7^ ^]^ Of interest, TNBC stem‐like cells exhibit hyperactive mitochondrial oxidative phosphorylation (OXPHOS), which is a biochemical process that occurs in the mitochondrial inner‐membrane of eukaryotic cells and activates the hypoxia pathway to maintain cancer cell stemness.^[^ ^8^, ^9^ ^]^ We and others recently demonstrated that OXPHOS is one of the most upregulated metabolic pathways in TNBC compared to normal mammary tissue,^[^ ^10^ ^]^ and is critical for survival, progression, and therapy resistance in TNBC.^[^ ^8^, ^11^, ^12^ ^]^ Consistently, residual TNBC cells surviving DNA‐damaging chemotherapies have increased mitochondrial fusion and OXPHOS.^[^ ^9^ ^]^ Thus, mitochondrial metabolism represents an attractive target for TNBC therapy.^[^ ^13^, ^14^ ^]^

Human transmembrane protein 65 (TMEM65) is a poorly characterized mitochondrial inner‐membrane protein.^[^ ^15^ ^]^ Available evidence suggests that TMEM65 regulates mitochondrial calcium efflux^[^ ^16^ ^]^ and the mitochondrial fusion process.^[^ ^17^ ^]^ Notably, depletion or mutation of TMEM65 leads to an induction of mitochondrial unfolded protein response^[^ ^18^ ^]^ and mitochondrial myopathy with severe neurological manifestations.^[^ ^19^ ^]^ By analyzing our recently published TNBC datasets of proteomics and transcriptomics,^[^ ^20^, ^21^ ^]^ we interestingly found that TMEM65 is upregulated in TNBC, and its high expression is associated with poor patient survival. However, the biological function, mechanism of action, and regulatory mechanism of TMEM65 in breast cancer including TNBC remain unexplored.

Serpin family B member 3 (SERPINB3), also known as squamous cell carcinoma antigen 1 (SCCA1), is a highly conserved serine/cysteine protease inhibitor and plays critical roles in various physiological and pathological processes, such as cell death protection and oncogenesis.^[^ ^22^, ^23^ ^]^ Interestingly, emerging evidence indicates that SERPINB3 expression is elevated in breast cancer cell lines and tissues,^[^ ^24^ ^]^ and its high expression is associated with advanced stage, high grade, and a decreased overall survival and recurrence‐free survival of breast cancer patients.^[^ ^24^ ^]^ Moreover, high expression of SERPINB3 predicts a poor prognosis in breast cancer patients treated with neoadjuvant chemotherapy.^[^ ^25^ ^]^ In addition, SERPINB3 promotes oncogenic transformation and epithelial‐mesenchymal transition in mammary epithelial cells.^[^ ^26^ ^]^ Despite these advances, it remains elusive how it is regulated in breast cancer and whether it contributes to breast cancer cell stemness.

In this study, we first report that transcription factor MYC and DNA demethylase ten‐eleven translocation 3 (TET3) cooperate to upregulate TMEM65 in TNBC. Upregulated TMEM65 promotes TNBC progression and cisplatin resistance both in vivo and in vitro, and pharmacological inhibition or knockdown of MYC and TET3 attenuates TMEM65‐driven TNBC progression. Moreover, we found that TMEM65 activates the OXPHOS‐SERPINB3 pathway to enhance TNBC stemness, thus leading to TNBC progression and chemoresistance. Overall, these findings identify TMEM65 as a novel oncogene and potential therapeutic target of TNBC.

## 2 Results

### 2.1 TMEM65 is Aberrantly Upregulated in TNBC Tissues and Its High Expression Predicts Poor Prognosis of TNBC Patients

**Figure 1:**
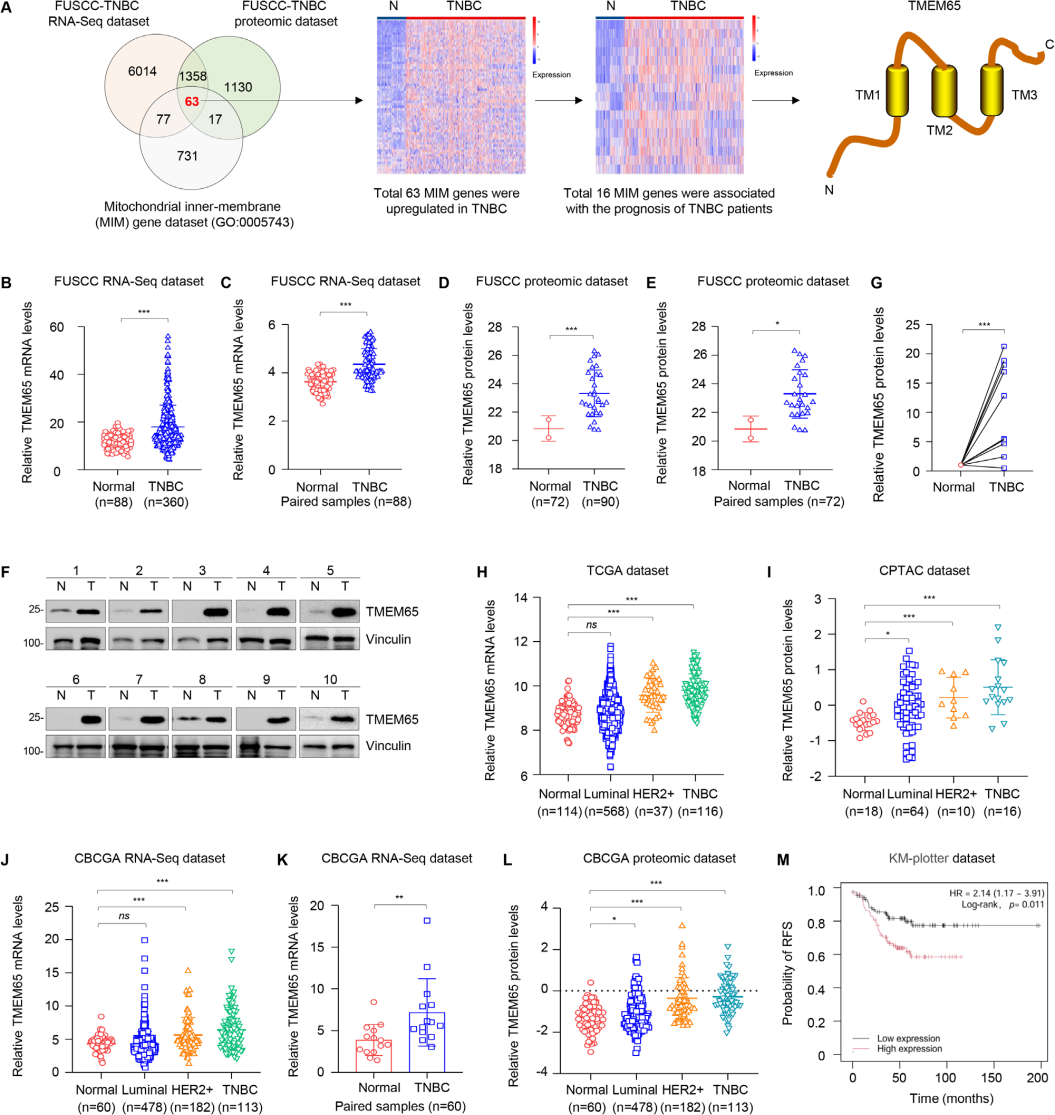
TMEM65 is aberrantly upregulated in TNBC tissues and its high expression predicts poor prognosis of TNBC patients. A) The schematic diagram for screening mitochondrial inner‐membrane (MIM)‐related genes in TNBC progression. TMEM65 protein contains three transmembrane (TM) domains. N, normal. B, C) The mRNA levels of TMEM65 in 360 TNBC tissues and 88 normal controls from the FUSCC‐TNBC dataset of RNA‐Seq.^[^ ^21^ ^]^ D, E) The protein levels of TMEM65 in 90 TNBC tissues and 72 normal controls from the FUSCC‐TNBC dataset of quantitative proteomics.^[^ ^20^ ^]^ F, G) Immunoblotting detection of TMEM65 protein expression levels in 10 pairs of primary TNBC specimens and adjacent normal tissues (F). The immunoblotting bands were quantified using ImageJ software, and the corresponding quantitative results are shown in G. H, I) The mRNA (H) and protein (I) levels of TMEM65 in all breast cancers from the TCGA (H) and CPTAC (I) datasets. J–L) The mRNA and protein levels of TMEM65 in all breast cancers from the CBCGA dataset.^[^ ^27^ ^]^ M) The association of TMEM65 mRNA levels with relapse‐free survival (RFS) of TNBC patients in the Kaplan–Meier plotter dataset (https://www.kmplot.com/analysis/).

OXPHOS occurs in the mitochondrial inner membrane (MIM) of eukaryotic cells with key roles in TNBC progression and therapeutic resistance and represents an attractive target for TNBC therapy.^[^ ^8^, ^9^, ^10^, ^11^, ^12^, ^13^, ^14^ ^]^ To identify novel MIM factors driving TNBC progression, we conducted integrative analyses of our recently published TNBC datasets of quantitative proteomics^[^ ^20^ ^]^ and RNA‐sequencing (RNA‐Seq)^[^ ^21^ ^]^ and MIM gene dataset (GO:0005743) (**Figure** **1**A). The results showed that the expression levels of 63 MIM genes were upregulated in TNBC (Figure S1, Supporting Information). Of those, high expression of 16 MIM genes was negatively associated with relapse‐free survival (RFS) of TNBC patients (Figure S2, Supporting Information). Finally, we chose TMEM65 as the focus of this study according to the innovation and importance of this gene.

Interestingly, we found that both protein and mRNA levels of TMEM65 were upregulated in total and paired TNBC specimens relative to normal controls in FUSCC‐TNBC datasets of RNA‐Seq (Figure 1B,C) and quantitative proteomics (Figure 1D,E). To further verify these results, we collected 10 pairs of primary TNBC specimens and adjacent normal tissues to detect the protein expression levels of TMEM65 by immunoblotting. The results showed that TMEM65 protein levels were upregulated in TNBC samples compared to normal controls (Figure 1F,G). Furthermore, we analyzed the expression status of TMEM65 in TNBC in three publicly available datasets, including TCGA, CPTAC, and CBCGA.^[^ ^27^ ^]^ Consistently, both protein and RNA levels of TMEM65 were upregulated in TNBC tissues, and their expression levels were higher in TNBC relative to luminal‐ and HER2‐positive subtypes of breast cancer (Figure 1H–L). Moreover, the prognostic analysis revealed that high expression of TMEM65 was associated with shorter relapse‐free survival (RFS) of TNBC patients (Figure 1M; Figure S2, Supporting Information). Taken together, these results suggest that TMEM65 is upregulated in TNBC and its upregulation predicts poor prognosis of TNBC patients.

### 2.2 TMEM65 Promotes TNBC Cell Proliferation, Migration, and Invasion In Vitro and Xenograft Tumor Growth and Lung Metastatic In Vivo

**Figure 2:**
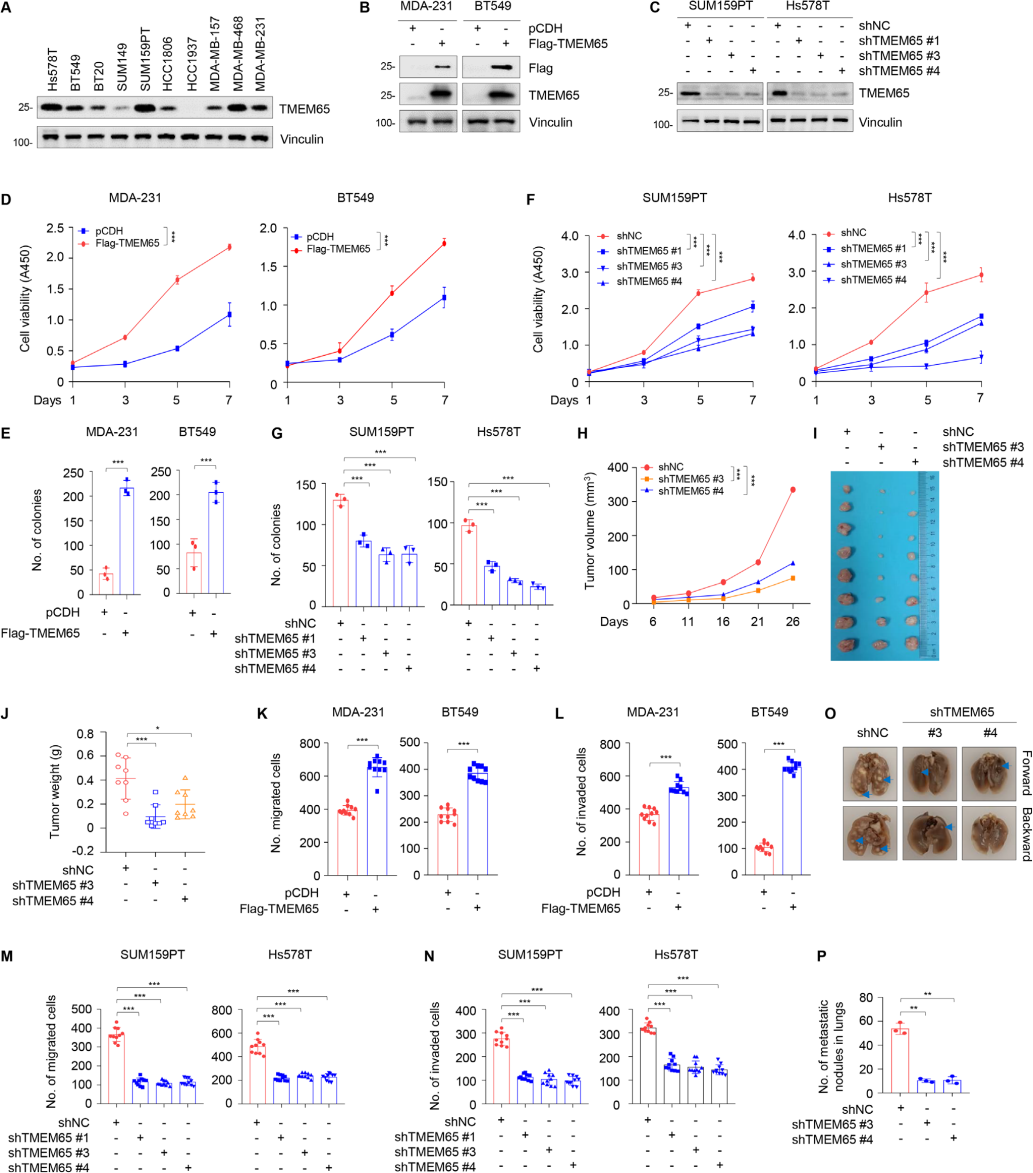
TMEM65 promotes TNBC cell proliferation, migration, and invasion in vitro and xenograft tumor growth and lung metastatic in vivo. A) Immunoblotting analysis of TMEM65 protein levels in 10 representative TNBC cell lines. B) Validation of the established MDA‐231 and BT549 cell lines stably expressing pCDH and Flag‐TMEM65 by immunoblotting. C) Validation of the established SUM159PT and Hs578T cell lines stably expressing shNC (negative‐control shRNA) and shTMEM65 (shRNA targeting TMEM65) by immunoblotting. D, E) MDA‐231 and BT549 cells stably expressing pCDH and Flag‐TMEM65 were subjected to CCK‐8 (D) and colony formation (E) assays. The representative images of survival colonies are shown in Figure S3A (Supporting Information). F, G) SUM159PT and Hs578T cells stably expressing shNC and shTMEM65 were subjected to CCK‐8 (F) and colony formation (G) assays. The representative images of survival colonies are shown in Figure S3B (Supporting Information). H–J) SUM159PT cells stably expressing shNC and shTMEM65 were injected into the mammary fat pad of female BALB/c nude mice to conduct xenograft tumor assays. Tumor growth rates were monitored for up to 26 days (H). The images of the collected xenograft tumors and tumor weight are shown in I and J, respectively. K, L) MDA‐231 and BT549 cells stably expressing pCDH and Flag‐TMEM65 were subjected to Transwell migration and invasion assays. The representative images of migrated and invaded cells are shown in Figure S3C (Supporting Information). M, N) SUM159PT and Hs578T cells stably expressing shNC and shTMEM65 were subjected to Transwell migration and invasion assays. The representative images of migrated and invaded cells are shown in Figure S3D (Supporting Information). O, P) SUM159PT cells stably expressing shNC and shTMEM65 were injected into the tail vein of female BALB/c nude mice to establish experimental lung metastasis models. The representative images of metastatic lung nodules and corresponding quantitative results are shown in O and P, respectively. The images of collected all lung tissues are shown in Figure S3E (Supporting Information).

To explore the biological functions of TMEM65 in TNBC progression, we first examined the protein expression levels of TMEM65 in 10 representative TNBC cell lines by immunoblotting (**Figure** **2**A). Based on the expression levels of TMEM65 in these cell lines, we stably overexpressed TMEM65 in MDA‐MB‐231 (thereafter named MDA‐231) and BT549 cells (Figure 2B), while knocked down endogenous TMEM65 in SUM159PT and Hs578T cells (Figure 2C) by lentiviral infection. The CCK‐8 and colony formation assays showed that ectopic expression of TMEM65 enhanced the proliferation and colony formation capability of MDA‐231 and BT549 cells (Figure 2D,E; Figure S3A, Supporting Information), whereas the opposite results were obtained in TMEM65‐depleted SUM159PT and Hs578T cells (Figure 2F,G; Figure S3B, Supporting Information). Importantly, the depletion of TMEM65 significantly reduced tumor volume and weight in mouse xenograft tumor models (Figure 2H–J).

Given the highly invasive and metastatic potential of TNBC cells, we then examined whether TMEM65 affects the migratory and invasive capacities of TNBC cells. Transwell migration and invasion assays showed that overexpression of TMEM65 enhanced the migratory and invasive potential of MDA‐231 and BT549 cells (Figure 2K,L; Figure S3C, Supporting Information), whereas knockdown of TMEM65 reduced the migratory and invasive potential of SUM159PT and Hs578T cells (Figure 2M,N; Figure S3D, Supporting Information). To confirm these results, we utilized experimental lung metastasis models by tail‐vein injection of TNBC cells into mice to examine the effects of TMEM65 on TNBC metastasis in vivo. As expected, there were fewer metastatic nodes in mice bearing TMEM65‐depleted tumors compared to corresponding controls (Figure 2O,P; Figure S3E, Supporting Information). These results were also confirmed by hematoxylin‐eosin (HE) staining of removed lung tissues from mice (Figure S3F, Supporting Information). Together, these results suggest that TMEM65 promotes TNBC progression both in vitro and in vivo.

### 2.3 Transcription Factor MYC and DNA Demethylase TET3 Cooperate to Transactivate TMEM65 in TNBC

To investigate the upstream regulatory mechanism of TMEM65 in TNBC, we first used the JASPAR program (https://jaspar.elixir.no/)^[^ ^28^ ^]^ to predict transcription factor binding profiles on the TMEM65 promoter. The results showed that the TMEM65 promoter region (−2000 to +200 bp) contains multiple putative binding motifs of transcription factor MYC (Figure S4A,B, Supporting Information), indicating that MYC may be an upstream transcription factor for TMEM65 in TNBC. We were interested in MYC because it is amplified in about 40% of TNBC and is causally associated with disease progression, therapeutic resistance, and poor outcomes in TNBC patients.^[^ ^8^, ^29^ ^]^

**Figure 3:**
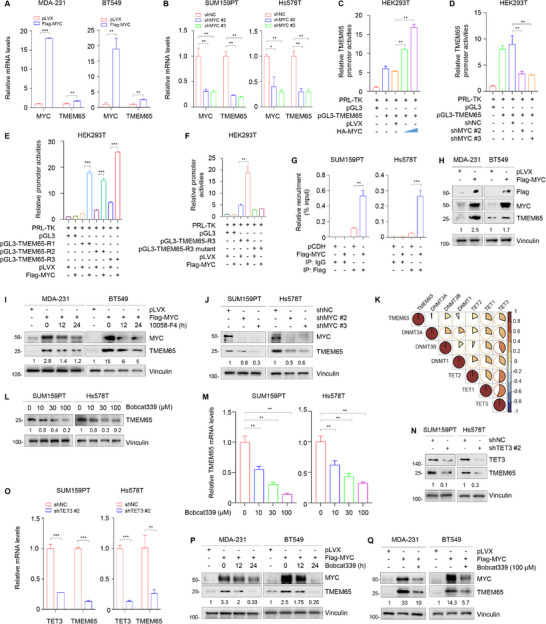
Transcription factor MYC and DNA demethylase TET3 cooperate to transactivate TMEM65 in TNBC. A, B) TNBC cells with ectopic expression (A) or knockdown (B) of MYC were subjected to RT‐qPCR assays to detect the mRNA levels of TMEM65. C, D) HEK293T cells with ectopic expression (C) or knockdown (D) of MYC were subjected to double luciferase reporter assays to detect TMEM65 promoter activities. E, F) HEK293T cells were transfected with the indicated expression vectors and subjected to luciferase assays to detect TMEM65 promoter activities. G) Recruitment of MYC to TMEM65 promoter was detected by ChIP assays. H, I) MDA‐231 and BT549 cells stably expressing pLVX and Flag‐MYC were treated without (H) or with MYC inhibitor 10058‐F4 (I) and were subjected to an immunoblotting assay to detect the protein levels of TMEM65. J) SUM159PT and Hs578T cells stably expressing shNC and shMYC were subjected to immunoblotting assays to detect the protein levels of TMEM65. K) The correlation of expression levels between the DNA methylation‐related enzymes and TMEM65 in the TCGA dataset. L, M) SUM159PT and Hs578T cells were treated with or without increasing doses of TET inhibitor Bobcat339 for 24 h, and then subjected to immunoblotting (L) and RT‐qPCR (M) assays to detect the protein and mRNA levels of TMEM65. N, O) SUM159PT and Hs578T cells stably expressing shNC and shTET3 were subjected to immunoblotting (N) and RT‐qPCR (O) assays to detect the protein and mRNA levels of TMEM65. P, Q) MDA‐231 and BT549 cells stably expressing pLVX and Flag‐MYC were treated without or with TET inhibitor Bobcat339, and then subjected to immunoblotting assays to detect the protein levels of TMEM65.

Indeed, RT‐qPCR assays showed that ectopic expression of MYC in MDA‐231 and BT549 cells upregulated TMEM65 mRNA levels (**Figure** **3**A), which were downregulated following knockdown of MYC in SUM159PT and Hs578T cells (Figure 3B). Consistently, the expression levels of MYC were positively correlated with TMEM65 expression levels in the FUSCC‐TNBC RNA‐Seq dataset^[^ ^21^ ^]^ (Figure S4C, Supporting Information). Luciferase assays demonstrated that overexpression of MYC increased (Figure 3C), whereas knockdown of MYC decreased (Figure 3D), the promoter activities of TMEM65. According to the score of the predicted binding sites of MYC on the TMEM65 promoter, we then divided the TMEM65 promoter (‐2000 to +200 bp) into 3 regions (R) (Figure S4D, Supporting Information). Luciferase assays showed that MYC significantly enhanced the promoter activities of TMEM65 at region 3 (−33 to +124 bp) (Figure 3E). Notably, mutation of the binding motif of MYC on the TMEM65 promoter significantly attenuated the promoter activities of TMEM65 under the condition of ectopic expression of MYC (Figure 3F). To further these results, we conducted ChIP assays with an anti‐MYC antibody or IgG as a control and then performed qPCR using primers designed against the region (−33 to +124 bp) of the TMEM65 promoter. Indeed, MYC was recruited to the tested promoter region of TMEM65 (Figure 3G). Consistently, ectopic expression of MYC resulted in an upregulation of TMEM65 protein levels (Figure 3H), and these effects were impaired in the presence of small‐molecule MYC inhibitor 10058‐F4^[^ ^30^ ^]^ (Figure 3I). Consistently, knockdown of MYC led to a downregulation of TMEM65 protein levels (Figure 3J). Together, these results suggest that MYC acts as an upstream transcription factor to transactivate TMEM65 in TNBC cells.

In addition, DNA methylation is a critical epigenetic mechanism of gene transcription.^[^ ^31^ ^]^ The DNA methylation levels are controlled by the concerted action of DNA methyltransferases (DNMT1, DNMT3A, and DNMT3B) and ten‐eleven translocation (TET) family proteins (TET1, TET2, and TET3), which catalyze methylation and demethylation of DNA, respectively.^[^ ^31^, ^32^ ^]^ Then, we examined the correlation of expression levels between the DNA methylation‐related enzymes and TMEM65 in the TCGA dataset and found that TET3 had the strongest correlation with TMEM65 (Figure 3K). Analysis of the public TCGA and CBCGA datasets revealed that TET3 expression levels were higher in TNBC relative to luminal‐ and HER2+ types of breast cancer (Figure S5A,B, Supporting Information). In addition, the expression levels of TET3 were positively correlated with TMEM65 expression levels in the FUSCC‐TNBC RNA‐Seq dataset^[^ ^21^ ^]^ (Figure S5C, Supporting Information). Moreover, treatment of SUM159PT and Hs578T cells with the TET inhibitor Bobcat339^[^ ^32^, ^33^ ^]^ decreased both protein (Figure 3L) and mRNA (Figure 3M) levels of TMEM65. Consistently, knockdown of TET3 downregulated both protein (Figure 3N) and mRNA (Figure 3O) levels of TMEM65.

To examine whether TET3 mediates DNA demethylation in the TMEM65 promoter region, the TMEM65 promoter around the MYC‐bound region was amplified by PCR using three different pairs of primers and then subjected to pyrosequencing for analyzing the relative methylation levels of TMEM65 promoter following knockdown of TET3 in SUM159PT and Hs578T cells. The results showed that the relative methylation levels of the TMEM65 promoter were increased by about 3.5%–21% following knockdown of TET3 at the three tested regions (R) in both SUM159PT and Hs578T cells (Figure S5D, Supporting Information). This data suggests that TET3 indeed induces DNA demethylation in the TMEM65 promoter region. In line with our results, previous studies have demonstrated that TET3 regulates its target gene expression by inducing promoter demethylation in other model systems.^[^ ^34^, ^35^, ^36^ ^]^ Collectively, these results indicate that TET3‐mediated DNA demethylation contributes to TMEM65 transcription.

We next examined whether MYC and TET3 coordinatively upregulate TMEM65 in TNBC. As shown in Figure 3P,Q, ectopic expression of MYC resulted in an upregulation of TMEM65, and this effect was impaired in the presence of TET inhibitor Bobcat339 in TNBC cells. These results suggest that MYC and TET3 may cooperate to upregulate TMEM65 in MDA‐231 and BT549 cells. In support of this notion, ChIP‐qPCR assays showed that the knockdown of TET3 attenuated the recruitment of MYC to the TMEM65 promoter (Figure S6A, Supporting Information). Luciferase assays further revealed that MYC‐activated promoter activities of TMEM65 were impaired in TET3‐depleted cells compared to control cells (Figure S6B, Supporting Information). Moreover, RT‐qPCR assays demonstrated that MYC‐mediated upregulation of TMEM65 mRNA levels was also compromised following the knockdown of TET3 (Figure S6C, Supporting Information). Based on these results and the findings from other studies,^[^ ^35^ ^]^ we proposed that TET3‐mediated DNA demethylation enhances chromatin accessibility at TMEM65 promoter for MYC binding to transactivate TMEM65 expression in TNBC cells.

To further investigate whether there is a functional interaction between TET3 and MYC, we conducted reciprocal IP assays using an anti‐MYC or anti‐TET3 antibody to examine the interaction between MYC and TET3 at the endogenous level. As shown in Figure S7A,B (Supporting Information), we found that endogenous MYC interacted with endogenous TET3 in SUM159PT and Hs578T cells. Immunoblotting assays showed that overexpression or knockdown of MYC did not significantly alter total protein expression levels of TET3 in SUM159PT and Hs578T cells (Figure S7C,D, Supporting Information). Consistently, previous studies have shown that MYC does not affect total protein expression levels of TET3, but activates the demethylase activities of TET3 by enhancing its nuclear accumulation.^[^ ^37^, ^38^ ^]^ We next examined the effects of knockdown or pharmacological inhibition of TET3 on the protein expression levels of MYC. It is worth mentioning that TET inhibitor Bobcat339 not only inhibits the catalytic activities of TET enzymes,^[^ ^32^, ^33^ ^]^ but also induces TET3 degradation in human and mouse cells.^[^ ^39^, ^40^ ^]^ As shown in Figure S7E,F (Supporting Information), knockdown or pharmacological inhibition of TET3 resulted in a decrease in MYC protein levels. In addition, we also noticed that TET inhibitor Bobcat339 downregulated the expression levels of ectopic expression of MYC in MDA‐231 and BT549 cells (Figure 3P,Q). In support of our results, it has been documented that TET3 positively regulates MYC expression in liver Kupffer cells^[^ ^41^ ^]^ and glioma stem cells.^[^ ^42^ ^]^ Collectively, these results, along with those from previous studies,^[^ ^37^, ^38^, ^41^, ^42^ ^]^ suggest that there is indeed a functional interaction between TET3 and MYC. Thus, we proposed that MYC and TET3 could form a positive loop to drive TMEM65 expression in TNBC cells.

### 2.4 Pharmacological Inhibition or Knockdown of MYC and TET3 Decelerates TMEM65‐Driven TNBC Progression

**Figure 4:**
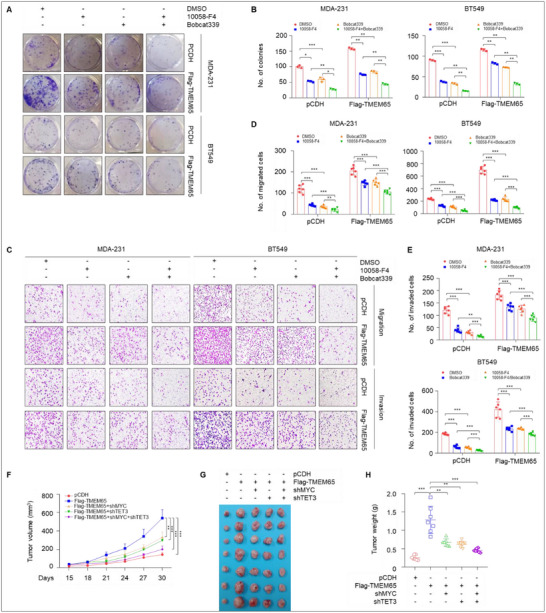
Pharmacological inhibition or knockdown of MYC and TET3 decelerates TMEM65‐driven TNBC progression. A, B) MDA‐231 and BT549 cells stably expressing pCDH and Flag‐TMEM65 were treated with or without MYC inhibitor 10058‐F4 or TET inhibitor Bobcat339 alone or in combination, and then subjected to colony formation assays. The representative images of survival colonies and corresponding quantitative results are shown in A and B, respectively. C–E) MDA‐231 and BT549 cells stably expressing pCDH and Flag‐TMEM65 were treated with or without MYC inhibitor 10058‐F4 or TET inhibitor Bobcat339 alone or in combination, and then subjected to Transwell migration and invasion assays. The representative images of migrated and invaded cells (C) and corresponding quantitative results (D, E) are shown. F–H) MDA‐231 cells stably expressing Flag‐TMEM65, shMYC, and shTET3 alone or in combination were injected into the mammary fat pad of female BALB/c nude mice to conduct xenograft tumor assays. Tumor growth rates were monitored for up to 30 days (F). The images of the collected xenograft tumors and tumor weight are shown in G and H, respectively.

As MYC and TET3 coordinatively transactivate TMEM65 and promote TNBC progression (Figures 2 and 3), we next investigated the possibility that pharmacological inhibition of MYC and TET could attenuate TMEM65‐driven TNBC progression. The results showed that treatment of cells with MYC inhibitor 10058‐F4 or TET inhibitor Bobcat339 alone reduced TMEM65‐induced colony formation capacity (**Figure** **4**A,B) and migratory and invasive potential (Figure 4C–E). Moreover, these effects were more pronounced in cells treated with 10058‐F4 combined with Bobcat339 (Figure 4A–E).

To avoid the potentially toxic effects of the combination administration of 10058‐F4 and Bobcat339 on mice, we stably knocked down MYC, TET3 alone or in combination in TMEM65‐overexpressing MDA‐231 cells and then carried out mouse xenograft tumor assays. The results showed that knockdown of MYC or TET3 alone significantly attenuated TMEM65‐induced xenograft tumor growth, and the more pronounced inhibitory effects were observed following simultaneous knockdown of MYC and TET3 (Figure 4F–H). These results further demonstrated that MYC and TET3 are upstream regulatory factors for TMEM65 in TNBC.

### 2.5 TMEM65 Transactivates Oncogene SERPINB3 Through Activating OXPHOS‐ROS‐HIF1α Pathway

**Figure 5:**
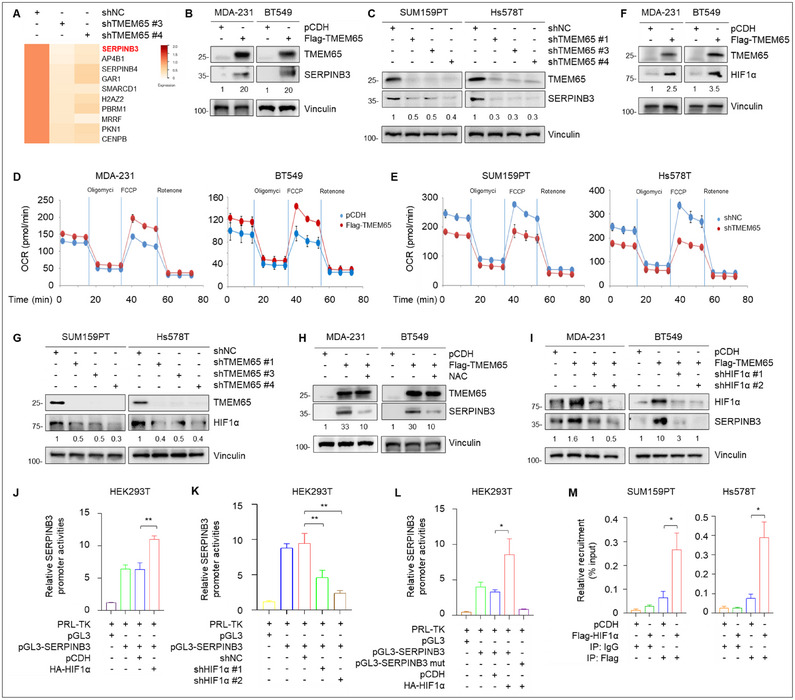
TMEM65 transactivates the oncogene SERPINB3 by activating the OXPHOS‐ROS‐HIF1α pathway. A) SUM159PT cells stably expressing shNC and shTMEM65 were subjected to label‐free quantitative proteomic assays. The top 10 down‐regulated proteins following the knockdown of TMEM65 are shown. B, C) Immunoblotting analysis of SERPINB3 protein expression levels under the conditions of ectopic expression (B) or knockdown (C) of TMEM65. D, E) The oxygen consumption rate (OCR) was determined in TNBC cells with ectopic expression (D) or knockdown (E) of TMEM65. F, G) Immunoblotting analysis of HIF1α protein expression levels in TNBC cells with ectopic expression (F) or knockdown (G) of TMEM65. H) MDA‐231 and BT549 cells stably expressing pCDH and Flag‐TMEM65 were treated with or without ROS inhibitor NAC for 48 h, and then subjected to immunoblotting assays to detect SERPINB3 protein expression levels. I) MDA‐231 and BT549 cells stably expressing pCDH, Flag‐TMEM65, and shHIF1α alone or in combination were subjected to immunoblotting assays to detect SERPINB3 protein expression levels. J–L) HEK293T cells were transfected with the indicated expression vectors and subjected to luciferase assays to detect TMEM65 promoter activities. M) Recruitment of HIF1α to SERPINB3 promoter was determined by ChIP assays.

To investigate the downstream mechanism of action of TMEM65 in TNBC progression, we carried out label‐free quantitative proteomic assays using SUM159PT cells stably expressing shNC and shTMEM65 (#3 and #4) (Figure S8A, Supporting Information). The results showed that 25 proteins were downregulated, while 15 proteins were upregulated following the knockdown of TMEM65 according to the cutoff value of 4‐fold change (Figure S8B, Supporting Information). The top 10 down‐regulated and up‐regulated proteins are shown in **Figures** **5**A and S8C (Supporting Information), respectively. KEGG (Kyoto Encyclopedia of Genes and Genomes) pathway analysis revealed that these differentially expressed proteins were mainly involved in cancer‐related pathways (Figure S8D, Supporting Information).

Previous studies have shown that SERPINB3 is a multifaceted oncogene involved in regulating cancer stemness,^[^ ^43^, ^44^ ^]^ epithelial‐mesenchymal transformation,^[^ ^26^ ^]^ tumor immunity,^[^ ^45^ ^]^ and cancer‐related inflammation,^[^ ^46^ ^]^ and is closely related to the progression of multiple types of human cancer.^[^ ^22^, ^23^ ^]^ Thus, we chose the top‐ranked downregulated protein SERPINB3 following knockdown of TMEM65 for further validation. Consistent with proteomic results, we found that ectopic expression of TMEM65 upregulated (Figure 5B; Figure S9A, Supporting Information), whereas knockdown of TMEM65 downregulated (Figure 5C; Figure S9B, Supporting Information), both protein and mRNA levels of SERPINB3. These results suggest that TMEM65 positively regulates SERPINB3 expression, at least in part, at the transcription level.

TMEM65 is mainly localized in the mitochondrial inner membrane,^[^ ^15^ ^]^ where OXPHOS occurs. In addition, OXPHOS promotes the production of reactive oxygen species (ROS),^[^ ^47^ ^]^ which in turn induces the expression of hypoxia‐inducible factor 1α (HIF1α).^[^ ^8^, ^48^ ^]^ As OXPHOS consumes more than 90% of oxygen in the body, we first detected the oxygen consumption rate (OCR) to measure mitochondrial OXPHOS.^[^ ^49^ ^]^ The results showed that ectopic expression of TMEM65 increased, whereas knockdown of TMEM65 decreased, the levels of OCR (Figure 5D,E). Consistently, we found that overexpression of TMEM65 resulted in an increase in ROS production and HIF1α expression (Figure 5F; Figure S9C, Supporting Information), whereas the opposite results were obtained following knockdown of TMEM65 (Figure 5G; Figure S9D, Supporting Information). These results collectively suggest that TMEM65 enhances mitochondrial OXPHOS, ROS production, and HIF1α expression in TNBC cells.

We next investigated whether TMEM65 transcriptionally regulates SERPINB3 by activating the OXPHOS‐ROS‐HIF1α pathway. Interestingly, we found that inhibition of ROS production by ROS inhibitor N‐acetylcysteine (NAC)^[^ ^50^ ^]^ or knockdown of HIF1α compromised TMEM65‐induced SERPINB3 expression (Figure 5H,I, respectively). Moreover, the mRNA levels of SERPINB3 were also decreased following knockdown of HIF1α in TMEM65‐overexpressing cells (Figure S9E, Supporting Information).

To further investigate how HIF1α affects SERPINB3 expression, we predicted the binding motifs of HIF1α on the SERPINB3 promoter using the transcription factor binding site database JASPAR (Figure S9F, Supporting Information). According to the score of the predicted binding sites of HIF1α on the SERPINB3 promoter (Figure S9G, Supporting Information), we cloned the SERPINB3 promoter (−1100 to +100 bp) and then conducted luciferase assays. The results showed that ectopic expression of HIF1α increased, whereas knockdown of HIF1α decreased, the promoter activities of SERPINB3 (Figure 5J,K, respectively). Moreover, mutation of the binding motif of HIF1α on the SERPINB3 promoter significantly attenuated SERPINB3 promoter activities following ectopic expression of HIF1α (Figure 5L). ChIP‐qPCR assays also demonstrated that HIF1α was recruited onto the SERPINB3 promoter (Figure 5M). Together, these results suggest that TMEM65 transactivates oncogene SERPINB3 by activating the OXPHOS‐ROS‐HIF1α pathway.

### 2.6 TMEM65 Promotes TNBC Progression Partially Via Regulating SERPINB3

**Figure 6:**
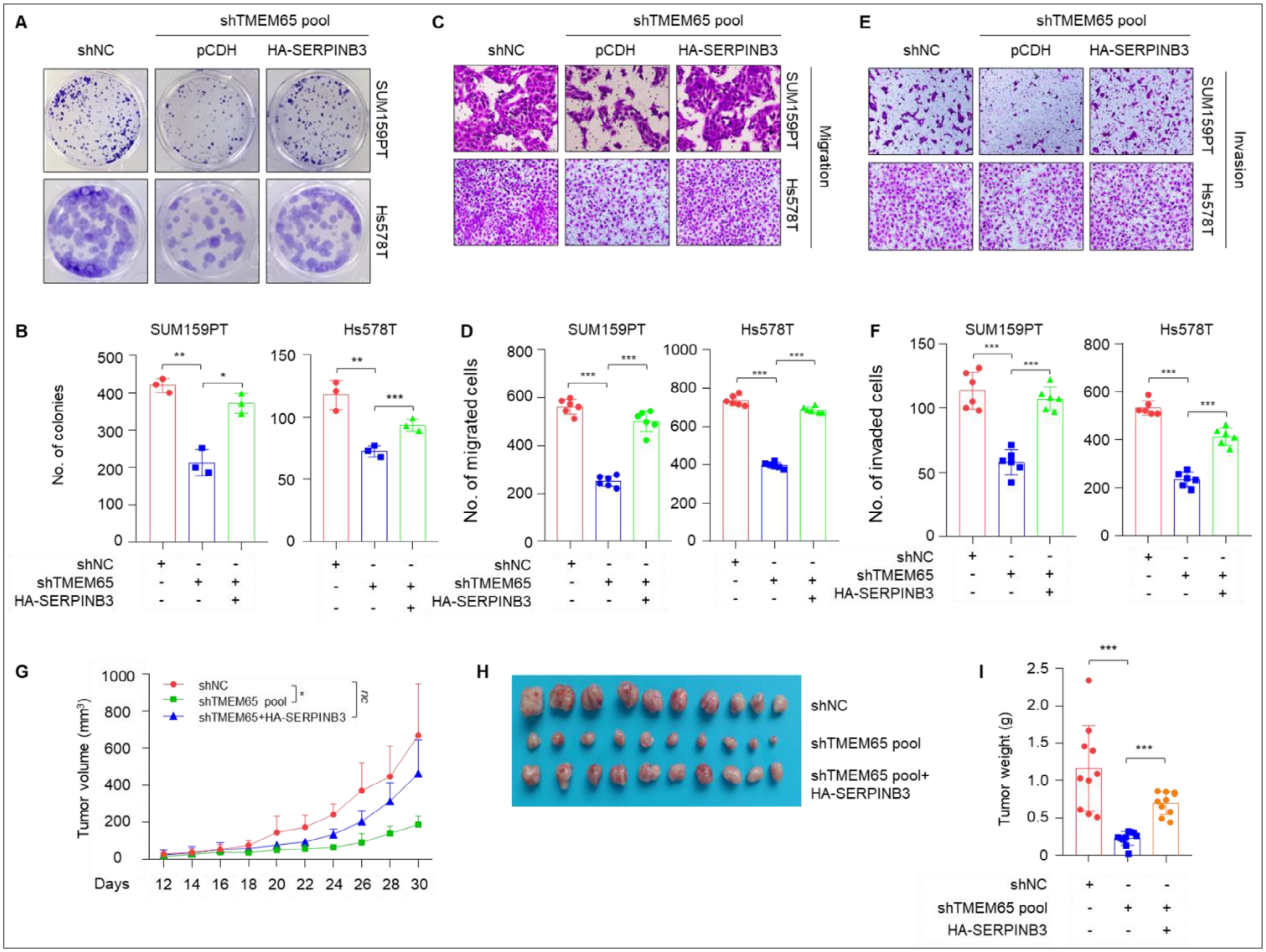
TMEM65 promotes TNBC progression partially via regulating SERPINB3. A, B) SUM159PT and Hs578T cells stably expressing shNC, shMYC, and HA‐SERPINB3 alone or in combination were subjected to colony formation assays. The representative images of survival colonies and corresponding quantitative results are shown in A and B, respectively. C–F) SUM159PT and Hs578T cells stably expressing shNC, shMYC, and HA‐SERPINB3 alone or in combination were subjected to Transwell migration (C, D) and invasion (E, F) assays. The representative images of migrated and invaded cells (C, E) and corresponding quantitative results (D. F) are shown. G–I) SUM159PT cells stably expressing shNC, shMYC, and HA‐SERPINB3 alone or in combination were subjected to mouse xenograft tumor assays. Tumor growth rates were monitored for up to 30 days (G). The images of the collected xenograft tumors and tumor weight are shown in H and I, respectively.

To examine whether TMEM65 promotes TNBC progression via regulating SERPINB3, we stably reintroduced SERPINB3 into TMEM65‐depleted SUM159PT and Hs578T cells (Figure S10, Supporting Information), and then we carried out in vitro experiments and xenograft tumor growth assays in vivo. The results showed that the re‐expression of SERPINB3 partially rescued the reduced colony formation capacity (**Figure** **6**A,B) and migratory and invasive potential (Figure 6C–F) of TNBC cells caused by knockdown of TMEM65. In vivo, xenograft tumor experiments also demonstrated that re‐expression of SERPINB3 partially restored the reduced xenograft tumor growth caused by knockdown of TMEM65 (Figure 6G–I). These results suggest that TMEM65 promotes TNBC progression partially via regulating SERPINB3.

### 2.7 TMEM65 Regulates TNBC Stemness via SERPINB3

**Figure 7:**
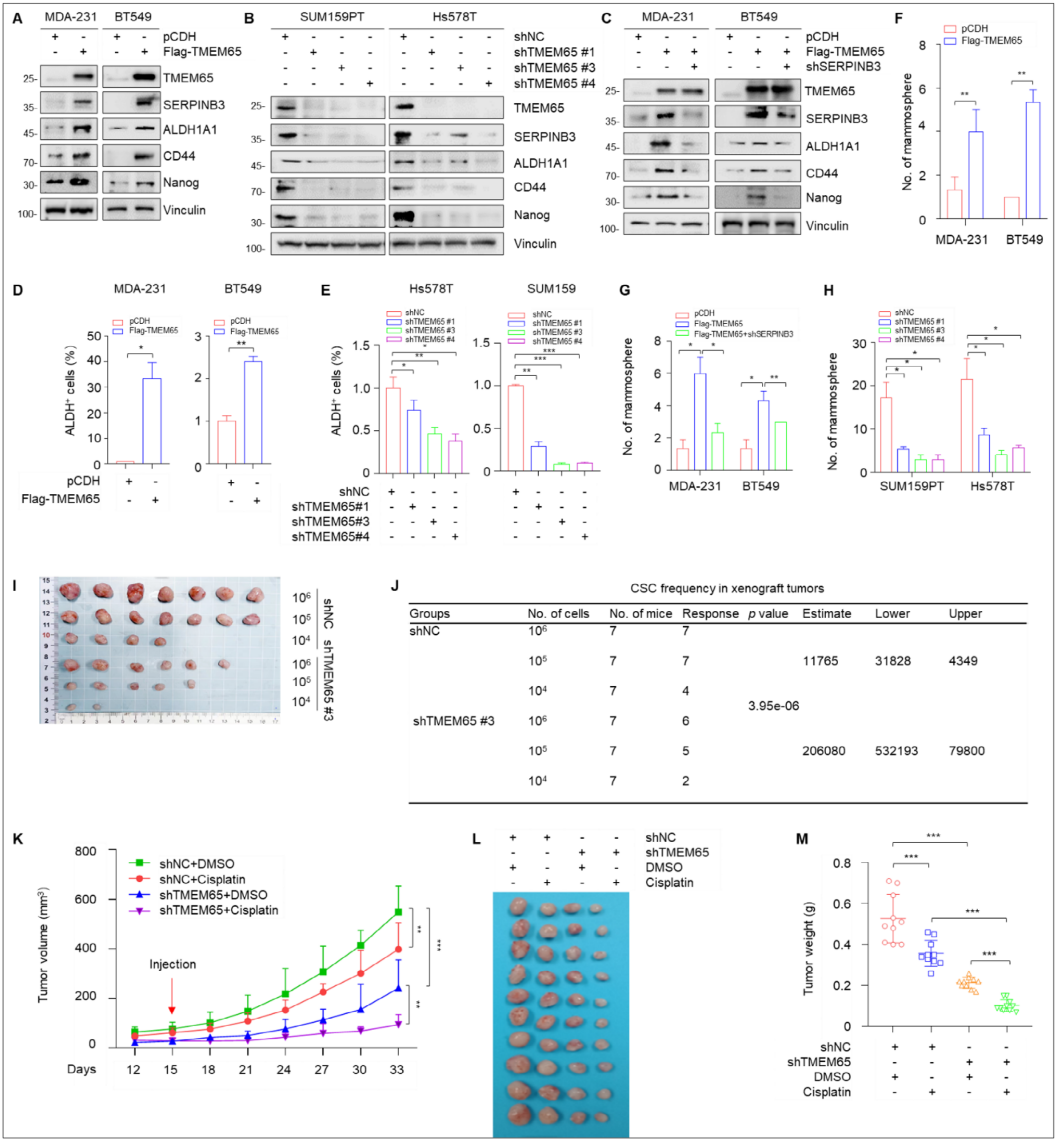
TMEM65 promotes TNBC stemness and cisplatin resistance both in vitro and in vivo. A, B) TNBC cells with ectopic expression (A) or knockdown (B) of TMEM65 were subjected to immunoblotting assays to detect the protein levels of SERPINB3 and putative CSC markers. C) MDA‐231 and BT549 cells stably expressing pCDH, Flag‐TMEM65, and shSERPINB3 alone or in combination were subjected to immunoblotting assays to detect the protein levels of SERPINB3 and putative CSC markers. D, E) TNBC cells with ectopic expression (D) or knockdown (E) of TMEM65 were subjected to ALDEFLUOR assays to detect the proportion of the putative CSC marker ALDH1‐positive cells. F, G) MDA‐231 and BT549T cells with ectopic expression of TMEM65 alone (F) or in combination with knockdown of SERPINB3 (G) were subjected to mammosphere assays to detect the sphere formation ability. H) SUM159PT and Hs578T cells with knockdown of TMEM65 were subjected to mammosphere assays to detect the sphere formation ability. I, J) SUM159PT cells stably expressing shNC and shTMEM65 were subjected to in vivo limiting dilution transplantation assays. The frequency of breast CSCs was calculated based on the positive tumor sites per group using ELDA (extreme limiting dilution analysis) software (http://bioinf.wehi.edu.au/software/elda/). K–M) SUM159PT cells stably expressing shNC and shTMEM65 were inoculated into the mammary fat pads of 6‐week‐old BALB/c female nude mice. After 15 days of injection, DMSO or cisplatin (3 mg kg^−1^) was administered to mice by intraperitoneal injection twice a week for 2 weeks. Tumor growth rates, the images of the collected xenograft tumors, and tumor weight are shown in K–M, respectively.

As it has been documented that SERPINB3 regulates cancer stemness,^[^ ^43^, ^44^ ^]^ we next examined whether TMEM65 affects TNBC stemness via SERPINB3. As expected, ectopic expression of TMEM65 increased (**Figure** **7**A), whereas knockdown of TMEM65 decreased (Figure 7B), the expression levels of SERPINB3 as well as the well‐documented cancer stem cell (CSC) markers including ALDH1A1 (aldehyde dehydrogenase 1 family member A1), CD44, and Nanog.^[^ ^51^ ^]^ Moreover, knockdown of SERPINB3 in TMEM65‐overexpressing cells attenuated TMEM65‐induced upregulation of ALDH1A1, CD44, and Nanog (Figure 7C). Consistently, ALDEFLUOR assays revealed that ectopic expression of TMEM65 enhanced (Figure 7D), whereas knockdown of TMEM65 reduced (Figure 7E), the proportion of the putative CSC marker ALDH1‐positive cells.

Then, we examined the effects of TMEM65 on the sphere formation ability of TNBC cells, which can, to a certain extent, reflect the self‐renewal ability of CSCs.^[^ ^52^ ^]^ The results showed ectopic expression of TMEM65 increased the formation of microspheres (Figure 7F), and the effects were attenuated when SERPINB3 was knocked down in TMEM65‐overexpersing cells (Figure 7G). In support of the notion that HIF1α transactivates SERPINB3, knockdown of HIF1α also attenuated TMEM65‐induced sphere formation ability (Figure S11A,B, Supporting Information). Consistently, knockdown of endogenous TMEM65 reduced the formation of microspheres in SUM159PT and Hs578T cells (Figure 7H).

The ability of CSCs to form tumors at low densities is one important test for determining prospective CSC population.^[^ ^52^ ^]^ In vivo limiting dilution assays, as the gold standard commonly used to estimate active CSC frequencies,^[^ ^52^ ^]^ revealed knockdown of TMEM65 compromised the potential tumor‐forming ability of TNBC cells in xenograft tumor assays (Figure 7I,J). Together, these results indicate that TMEM65 acts as a novel regulator of TNBC stemness partially via regulating SERPINB3.

### 2.8 TMEM65 Promotes Resistance of TNBC Cells to Cisplatin Both In Vitro and In Vivo

As cancer stemness is intimately linked to chemoresistance in TNBC^[^ ^7^ ^]^ and cisplatin is a commonly used chemotherapy drug for TNBC patients,^[^ ^4^ ^]^ we next examined the effects of TMEM65 on the sensitivity of TNBC cells to cisplatin. CCK‐8 and colony formation assays showed that knockdown of TMEM65 enhanced cellular sensitivity to cisplatin (Figure S12A,C, Supporting Information). Consistently, mouse xenograft tumor assays also revealed that treatment of cisplatin suppressed xenograft tumor growth in mice bearing shNC‐expressing tumors, and the tumor suppressive effects of cisplatin were more pronounced in mice bearing shTMEM65‐expressing tumors (Figure 7K–M). These results suggest that TMEM65 contributes to cisplatin resistance in TNBC cells.

## 3 Discussion

In this study, we first report several interesting findings concerning the previously unrecognized functional and mechanistic roles of TMEM65 in TNBC progression and chemoresistance (Figure S13, Supporting Information).

First, TMEM65 acts as a novel oncogene in TNBC to promote cancer progression and cisplatin resistance. The transmembrane (TMEM) protein family is a group of membrane‐spanning proteins localized to the plasma membrane or intracellular organelle membranes.^[^ ^53^ ^]^ These proteins can serve as ion channels, signal receptors, transporters, and enzymes with crucial roles in cellular physiologic and pathological functions, and represent ideal drug targets.^[^ ^53^, ^54^, ^55^, ^56^, ^57^, ^58^ ^]^ Notably, about 15%–30% of human gene‐encoded proteins are predicted to be TMEM proteins.^[^ ^59^ ^]^ However, due to their complex structure and cellular localization and the difficulty in purifying these proteins, the structure, function, and mechanism of action of most TMEM proteins are currently unclear.^[^ ^53^, ^60^ ^]^ A case in point is human TMEM65, a poorly characterized mitochondria‐localized TMTM protein containing three transmembrane domains.^[^ ^15^ ^]^ Currently available evidence suggests that TMEM65 contributes to mitochondrial metabolism^[^ ^15^, ^16^, ^17^ ^]^ and is critical for the structure and function of the intercalated discs in the hearts.^[^ ^61^, ^62^ ^]^ Interestingly, two recent studies indicate that TMEM65 expression may be related to the development of gastric and colorectal cancer.^[^ ^17^, ^63^ ^]^ However, there is no literature about the functional and mechanistic role of TMEM65 in breast cancer including TNBC. In this study, we provide the first evidence that TMEM65 promotes TNBC cell proliferation, migration, and invasion in vitro and xenograft tumor growth and lung metastatic in vivo (Figure 2), and decreases TNBC cellular sensitivity to cisplatin in vitro and in mouse models (Figure 7). Thus, TMEM65 functions as a novel oncogene in TNBC and may represent a potential therapeutic target for TNBC therapy.

Second, MYC and TET3 act as upstream regulators of TMEM65 in TNBC, and pharmacological inhibition or knockdown of MYC and TET3 suppresses TMEM65‐driven TNBC progression. MYC is one of the most frequently altered driver genes in TNBC, which is amplified in about 40% of TNBC and drives multiple oncogenic gene expression programs to promote disease progression and therapeutic resistance.^[^ ^8^, ^29^, ^64^ ^]^ Consequently, targeting MYC using small‐molecule inhibitors or degraders has been shown to suppress TNBC progression.^[^ ^65^, ^66^ ^]^ In addition, DNA demethylation mediated by the TET family of proteins (TET1, TET2, and TET3) represents a crucial epigenetic modification that manipulates gene expression in numerous biological processes.^[^ ^67^ ^]^ Previous studies have shown that TET1 and TET3 are upregulated in breast cancer and correlate with an unfavorable patient prognosis.^[^ ^68^, ^69^, ^70^, ^71^ ^]^ In addition, hypoxia significantly increases the expression of TET1 and TET3 via HIF1α to promote stem cell‐like features in breast cancer cells.^[^ ^70^ ^]^ TET3‐mediated promoter hypomethylation leads to an upregulation of IGF2BP3 (insulin‐like growth factor 2 mRNA‐binding protein 3) expression to accelerate TNBC proliferation.^[^ ^72^ ^]^ Not surprisingly, TET inhibitor Bobcat339 blocks cytokine interleukin‐1β‐induced breast cancer progression and bone metastasis formation.^[^ ^73^ ^]^ In this study, we demonstrated that MYC and TET3 act as upstream regulators to coordinatively transactivate TMEM65 in TNBC (Figure 3). Consistently, pharmacological inhibition or knockdown of MYC and TET3 partially blocks TMEM65‐driven TNBC progression (Figure 4).

Third, TMEM65 acts as a novel regulator of TNBC stemness by activating the OXPHOS‐ROS‐HIF1α‐SERPINB3 pathway to promote cancer progression and chemoresistance. Accumulating evidence shows that CSCs are enriched in TNBC and are responsible for TNBC recurrence, metastasis, and chemoresistance.^[^ ^6^, ^7^ ^]^ Consequently, targeting CSCs represents an attractive therapeutic strategy for TNBC.^[^ ^74^ ^]^ In this study, we first demonstrated that TMEM65 acts as a novel regulator of TNBC stemness to promote multiple cancer stemness properties, including stemness markers expression, sphere formation, tumorigenicity with low numbers of cancer cell implantation in mice, and cisplatin resistance (Figures 5 and 7). Mechanistic investigations further revealed that SERPINB3 acts as a downstream transcriptional target of TMEM65 by label‐free quantitative mass spectrometry and multiple biochemical assays (Figure 5) and that TMEM65 transactivates SERPINB3 expression in TNBC cells by activating mitochondrial OXPHOS‐ROS‐HIF1α pathway (Figure 5).

In support of our findings, it has been shown that MYC, as an upstream regulatory regulator of TMEM65, is a key factor required for CSC survival and reprogramming in TNBC,^[^ ^8^, ^75^, ^76^ ^]^ and promotes chemoresistance in TNBC by expanding CSCs via activating OXPHOS‐ROS‐HIF1α pathway.^[^ ^8^ ^]^ TET3, as another upstream regulator of TMEM65, also contributes to the formation of CSCs, cancer progression, and chemoresistance.^[^ ^77^, ^78^, ^79^ ^]^ In addition, it has been documented that oncogene SERPINB3 is involved in regulating cancer stemness in human cholangiocarcinoma and glioblastoma,^[^ ^43^, ^44^ ^]^ and its high expression is associated with resistance to platinum‐based chemotherapy in non‐small‐cell lung cancer.^[^ ^80^ ^]^

Collectively, the findings presented here suggest that MYC/TET3‐regulated TMEM65 functions as a novel oncogene in TNBC to promote TNBC progression and cisplatin resistance by activating the OXPHOS‐SERPINB3 pathway. These emerging findings suggest that targeting TMEM65 is a promising strategy to suppress TNBC progression and sensitize TNBC to DNA‐damaging agent cisplatin. As a novel regulator of cancer stemness, whether elevated expression of TMEM65 contributes to therapeutic resistance to other chemotherapy agents (in addition to cisplatin) or targeted therapeutic drugs remains to be explored in future studies.

## 4 Experimental Section

### Cell Culture and Reagents

Human embryonic kidney 293T cell line (HEK293T) and 10 representative TNBC cell lines (MDA‐MB‐231, MDA‐MB‐468, MDA‐MB‐157, HCC1937, HCC1806, SUM149, SUM159PT, BT20, BT549, and Hs578T) were obtained from the Cell Bank of Chinese Academy of Sciences (Shanghai, China) and Shanghai Key Laboratory of Breast Cancer (Fudan University, Shanghai, China). Cells were cultured in DMEM medium (BasalMedia, #L110) containing 10% fetal bovine serum (ExCell Biol, #FSP500) and 1 × penicillin‐ streptomycin (BasalMedia, #S110B). All cells were cultured with a 5% CO_2_ incubator at 37 °C. Other chemical reagents used in this study are listed in Table S1 (Supporting Information).

### Clinical Samples and Datasets

TNBC samples and normal breast tissues were obtained from patients who underwent surgery in FUSCC (Fudan University Shanghai Cancer Center) without any treatment. All procedures were approved by the Medical Ethics Committee of FUSCC. TNBC datasets of quantitative proteomics^[^ ^20^ ^]^ (90 TNBC samples and 72 normal samples) and RNA‐Seq^[^ ^21^ ^]^ (360 TNBC samples and 88 normal samples) as well as the CBCGA (Chinese Breast Cancer Genome Atlas) dataset^[^ ^27^ ^]^ were obtained from the FUSCC cohort. In addition, both TCGA (The Cancer Genome Atlas) and CPTAC (Clinical Proteomic Tumor Analysis Consortium) datasets were downloaded from the cBioPortal website (https://www.cbioportal.org/).

### DNA Plasmids and Lentiviral Infection

The cDNAs of TMEM65, TET3, MYC, and HIF1α were amplified by PCR and then subcloned into the lentiviral vector pCDH‐CMV‐MCS‐EF1‐Puro (System Biosciences, #CD511B‐1). The primers used for molecular cloning are provided in Table S2 (Supporting Information). The specific short hairpin RNA (shRNA) sequences targeting TMEM65, TET3, MYC, and HIF1α were obtained from Sigma–Aldrich Advanced Genomics (Table S3, Supporting Information), synthesized at GenePharma (Shanghai, China), and subcloned into the lentiviral vector pLKO.1‐TRC (Addgene, #10878).

To generate stable cell lines, the indicated constructs in lentiviral expression vectors together with packaging plasmid mix were transfected into HEK293T cells. After 48 h of transfection, cell supernatants were collected and used for infecting cells when cell density reached 70%–80% confluence in the presence of 8 µg mL^−1^ of polybrene (Sigma–Aldrich, #H9268). After 48 h of infection, cells were selected with 2 µg mL^−1^ of puromycin (Sangon Biotech, #A610593) for about 1 week.

### RNA Extraction and RT‐qPCR Assays

Total RNA was isolated from cultured cells using RNA TRIzol reagent (ThermoFisher, #15596018), and cDNA was generated using PrimeScript RT Master Mix (Takara, #RR036A) according to the manufacturer's protocol. Real‐time‐quantitative PCR (RT‐qPCR) assays were conducted using SYBR Premix Ex Taq (Tli RNaseH Plus) (Takara, #RR420B). RT‐qPCR primers were synthesized at HuaGene Biotech (Shanghai, China), and the corresponding sequences are provided in Table S4 (Supporting Information). All experiments were repeated at least three times.

### Immunoblotting Assays

For immunoblotting assays, proteins were isolated using RIPA buffer and quantified using BCA reagent (Yeasen Biotech, #20201ES90). Then, proteins were isolated by SDS‐PAGE and then transferred to a PVDF membrane (Millipore, #IPVH00010). After being blocked for 2 h with 5% bovine serum albumin (Yeasen Biotech, #36101ES80) at room temperature, the membranes were then incubated with the indicated antibodies at 4 °C overnight. The protein bands were visualized using enhanced chemiluminescence (ECL) detection kit (Yeasen Biotech, #36208ES76) and quantified using ImageJ software (https://imagej.net/ij/). All antibodies used in this study are listed in Table S5 (Supporting Information).

### Luciferase Assays

The promoters of TMEM65 and SERPINB3 genes were cloned into luciferase reporter vector pGL3‐basic using the primers provided in Table S6 (Supporting Information). Luciferase assays were performed using a Dual‐Luciferase Reporter Gene Assay Kit (Yeasen Biotech, #11402ES60) according to the manufacturer's instructions.

### ChIP‐qPCR Assays

ChIP assays were performed using a SimpleChIP Enzymatic Chromatin IP Kit (Magnetic Beads) (Cell Signaling Technology, #9003S) according to the manufacturer's instructions, followed by qPCR assays using the listed primers in Table S7 (Supporting Information).

### Pyrosequencing Analysis

Analysis of DNA methylation by pyrosequencing had been described previously in detail.^[^ ^81^, ^82^ ^]^ Briefly, genomic DNA was extracted from cultured cells using the DNeasy Blood & Tissue Kit (Qiagen, # 69506) and then bisulfite was converted using the EpiTect Bisulfite Kit (Qiagen, #59104) according to the manufacturer's instructions. The converted DNA was utilized for PCR amplification of the TMEM65 promoter with the PyroMark PCR kit (Qiagen, # 978703). The PCR products were subjected to pyrosequencing by PyroMark Q48 instrument (Qiagen, #9002471) at Shanghai Geneland Biotech (Shanghai, China). The results were analyzed using the associated PyroMark Q48 software (Qiagen, # 9024325). The primers for PCR amplification and sequencing were designed using the PyroMark Assay Design 2.0 software (Qiagen, #9019077) and are listed in Table S8 (Supporting Information). The increased relative methylation levels (%) of TMEM65 promoter following TET3 knockdown were calculated by the following formula:

(1) [(shTET3−shNC)/shNC]×100%

### XF Cell Mito Stress Assays

The oxygen consumption rate (OCR) was detected using Seahorse XF Cell Mito Stress Test Kit (Agilent, #103015‐100) and Seahorse XFe96/XF Pro FluxPak Mini (Agilent, #103793‐100) according to the manufacturer's instructions.

### Measurement of Intracellular ROS Levels

Cells were suspended in a serum‐free medium containing reactive oxygen species (ROS) fluorescence probe DCFH‐DA (2, 7‐dichlorodihydrofluorescein diacetate) (MedChemExpress, #2044‐85‐1) for 60 min. The stained cells were washed twice with serum‐free medium and then subjected to flow cytometry analysis.

### ALDEFLUOR Assays

The ALDEFLUOR assays were performed using the ALDEFLUOR Kit (STEMCELL Tech, #01700) to identify cells expressing aldehyde dehydrogenase (ALDH) according to the manufacturer's instructions. The stained samples were analyzed by flow cytometry using a MoFlo Astrios instrument (Beckman Coulter, USA), and data analysis was performed using Summit software.

### Cell Counting Kit‐8 (CCK‐8) and Colony Formation Assays

For CCK‐8 assays, cells were plated in 96‐well plates and each experimental group had five replicates. CCK‐8 kit (Yeasen Biotech, #40203ES92) was used to detect cell proliferation rates according to the manufacturer's protocol. For colony formation assays, cells were plated in 6‐well plates. Each experimental group had three replicates. After 14 days of growth, cells were fixed using methanol and stained using 0.2% crystal violet.

### Cell Migration and Invasion Assays

For cell migration assays, 3 × 10^4^ cells in serum‐free medium were plated in the upper chamber without matrix (Corning Falcon, #353097). For cell invasion assays, 6 × 10^4^ cells in serum‐free medium were plated in the upper chamber with Matrix (Corning BioCoat, #354480). Medium with 10% FBS (800 µL) was added to the lower chamber. After about 24 h of incubation, cells in the lower chamber were fixed with methanol for 30 min and stained with 0.2% crystal violet.

### Mammosphere Assays

Sphere assays were performed as described previously.^[^ ^83^ ^]^ Briefly, cells were seeded into 96‐well ultra‐low attachment plates (Corning BioCoat, #4515) in serum‐free DMEM supplemented with 4 µg mL^−1^ heparin (STEMCELL Tech, #07980), 1 µg mL^−1^ hydrocortisone (Sigma–Aldrich, #H0135), and 1 × penicillin‐streptomycin, and cultured at 37 °C with 5% CO_2_. After 15 days, spheres were counted at × 40 magnification under a microscope.

### Xenograft Mouse Models and Treatment

All experimental procedures were approved by the Animal Ethics Committee of FUSCC. To evaluate the effects of TMEM65 and cisplatin on xenograft tumor growth, 1 × 10^6^ cells were inoculated into the mammary fat pad of 5‐week‐old BALB/c female nude mice. When the mean tumor volume reached 100 mm^3^, DMSO or cisplatin (3 mg kg^−1^) was injected intraperitoneally twice a week. The tumor size was measured every two days, and the formula for calculating tumor volume was (length × width^2^)/2. To evaluate the effects of TMEM65 and cisplatin on the pulmonary metastasis capacity of TNBC cells, 1 × 10^5^ cells were inoculated into 6‐week‐old female nude BALB/c mice by tail vein injection. During the experiments, once nude mice lost 20% of their body weight or developed cachexia, they were sacrificed using the cervical dislocation method, and xenograft tumors or lungs were removed from the mice.

### Statistical Analysis

All data were represented as mean ± standard deviation (SD) from at least three independent experiments. The GraphPad (version 8.0.2), SPSS (version 20.0), two‐tailed *t*‐test, or one‐way analysis of variance were used to conduct statistical analysis between the two groups. The Spearman test was used for performing correlation analysis. Survival curves were analyzed by the Kaplan–Meier method and log‐rank test. *P*<0.05 was considered statistically significant. (^*^, *p*<0.05; ^**^, *p*<0.01; ^***^, *p*<0.001; ns, no significance).

## Conflict of Interest

The authors declare no conflict of interest.

## Author Contributions

Y.‐L.Z. and M.‐Y.H. contributed equally to this work. Y.‐L.Z. did data curation, formal analysis, investigation, writing the manuscript draft, and funding acquisition. M.‐Y.H., S.‐Y.Y., J.‐Y.C., and Q.Z. did data analysis and investigation. F.‐L.Z. did Funding acquisition and technical assistance. Z.‐M.S. and A.‐Y.C. did clinical specimens and research resources. L.L. did data analysis and funding acquisition. D.‐Q.L. did funding acquisition, project administration, and manuscript editing.

## Supporting information



Supporting Information

## Data Availability

The data that support the findings of this study are available from the corresponding author upon reasonable request.
