# Supplementary material for: MYC/TET3‐Regulated TMEM65 Activates OXPHOS‐SERPINB3 Pathway to Promote Progression and Cisplatin Resistance in Triple‐Negative Breast Cancer
Source: Adv Sci (Weinh). 2025 Jun 23;12(34):e00421. doi: 10.1002/advs.202500421 (PMC12442607; doi:10.1002/advs.202500421)
Supplement: Supplementary file 1 — Supporting Information [file ADVS-12-e00421-s001.pdf]

ADVANCED SCIENCE
Open Access

Supporting Information

for *Adv. Sci.*, DOI 10.1002/advs.202500421

MYC/TET3-Regulated TMEM65 Activates OXPHOS-SERPINB3 Pathway to Promote Progression and Cisplatin Resistance in Triple-Negative Breast Cancer

Yin-Ling Zhang, Min-Ying Huang, Shao-Ying Yang, Jia-Yang Cai, Qian Zhao, Fang-Lin Zhang, Xin Hu, Zhi-Min Shao, Li Liao, A-Yong Cao* and Da-Qiang Li**

Supplementary information for

Zhang at al. MYC/TET3-Regulated TMEM65 Activates OXPHOS-SERPINB3 Pathway to Promote Progression and Cisplatin Resistance in Triple-Negative Breast Cancer

This supplementary information includes

Supplementary Figures: 13

Supplementary Tables: 8

Supplementary Figures and Figure legends

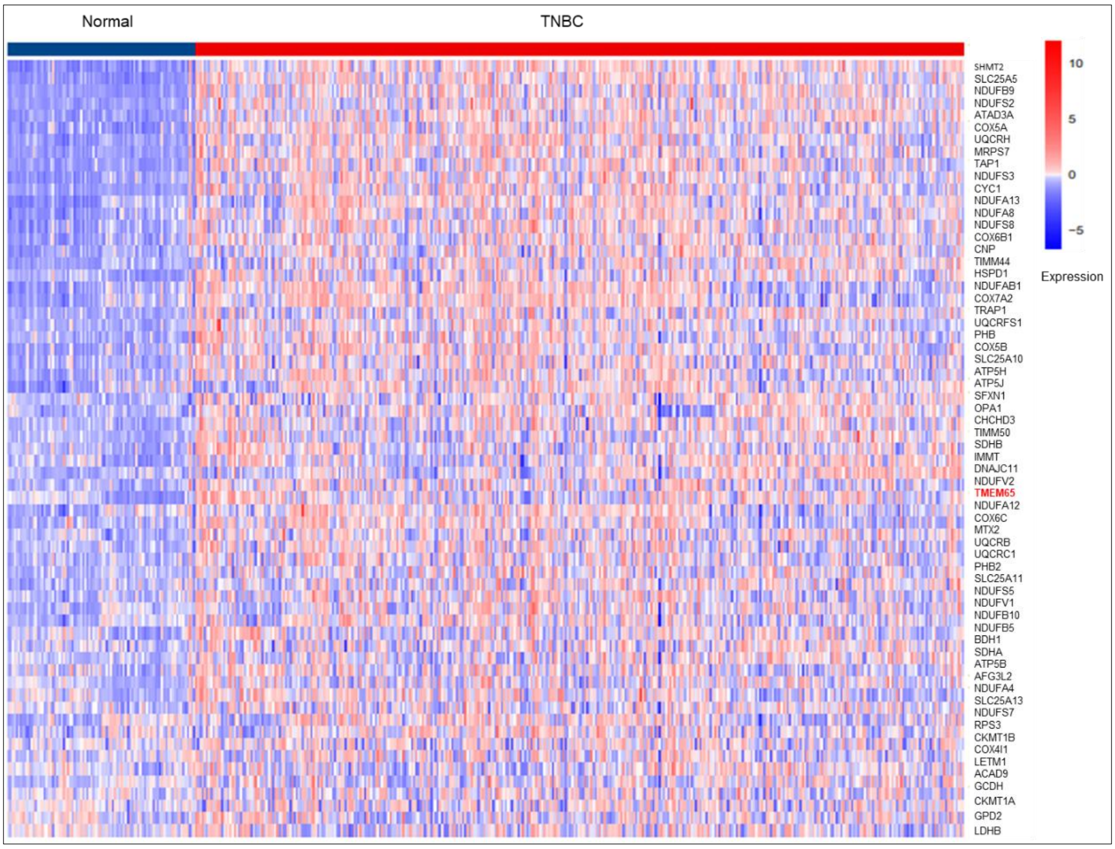

Supplementary Fig. S1. Screening of mitochondrial inner-membrane (MIM)-related genes in TNBC progression.

Heat map showing 63 MIM-related genes that were upregulated in TNBC.

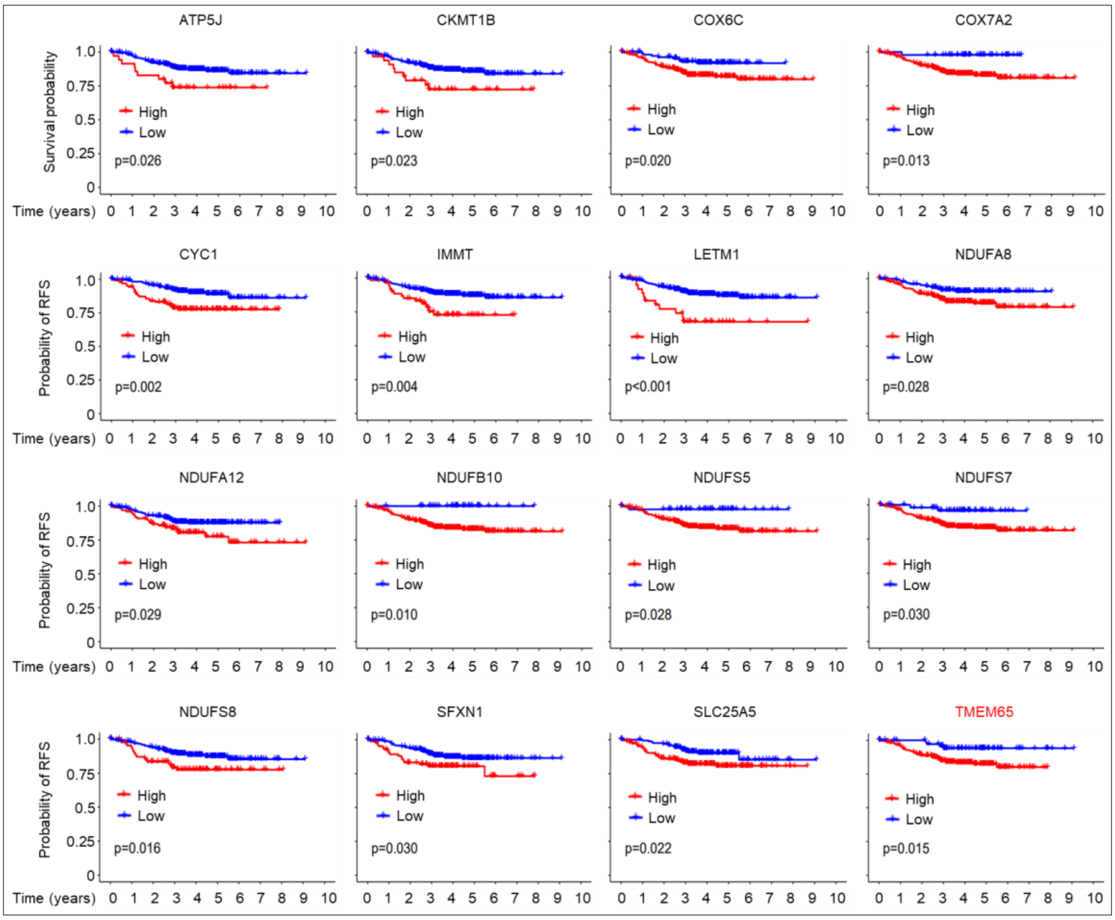

Supplementary Fig. S2. The association of the expression levels of 16 MIM-related genes with relapse-free survival (RFS) of TNBC patients in FUSCC-TNBC proteomic dataset.

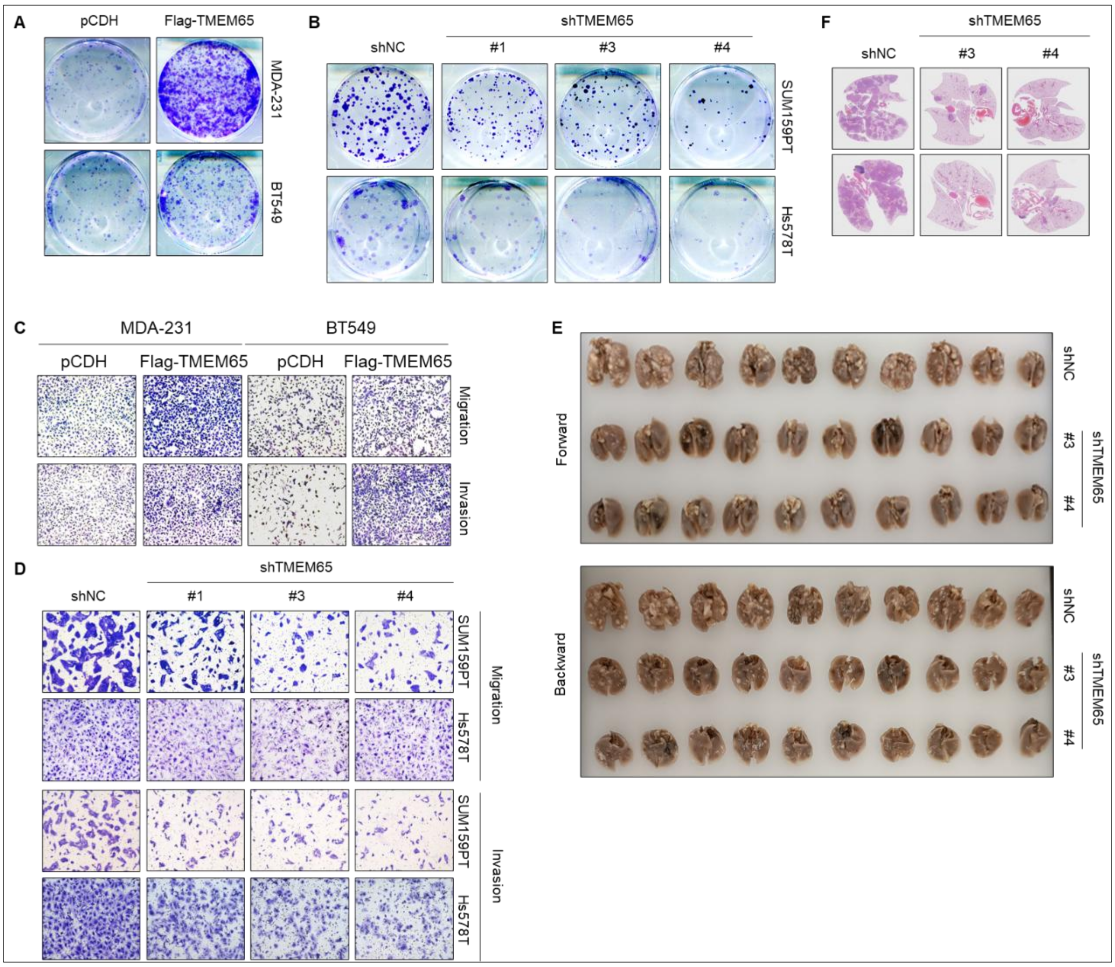

Supplementary Fig. S3. TMEM65 promotes TNBC cell proliferation, migration, and invasion *in vitro* and xenograft tumor growth and lung metastatic *in vivo*

(**A**) MDA-231 and BT549 cells stably expressing pCDH and Flag-TMEM65 were subjected to colony formation assays. The representative images of survival colonies are shown. The corresponding quantitative results are shown in Fig. 2E.

(**B**) SUM159PT and Hs578T cells stably expressing shNC and shTMEM65 were subjected to colony formation assays. The representative images of survival colonies are shown. The corresponding quantitative results are shown in Fig. 2G.

(**C**) MDA-231 and BT549 cells stably expressing pCDH and Flag-TMEM65 were subjected to Transwell migration and invasion assays. The representative images of migrated and invaded cells are shown. The corresponding quantitative results are shown in Figs. 2K-L, respectively.

(**D**) SUM159PT and Hs578T cells stably expressing shNC and shTMEM65 were subjected to Transwell migration and invasion assays. The representative images of migrated and invaded

cells are shown. The corresponding quantitative results are shown in Figs. 2M-N, respectively. (**E-F**) SUM159PT cells stably expressing shNC and shTMEM65 were injected into the tail vein of female BALB/c nude mice to establish experimental lung metastasis models. The images of collected lung tissues are shown (E). The representative images of hematoxylin-eosin (HE) staining of lung tissues from mice are shown in F.

A

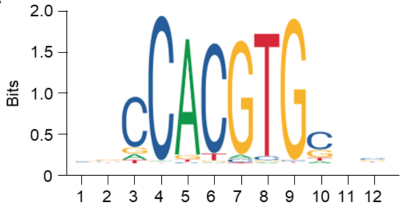

B

Sequence ID	Score	Relative score	Start	End
TMEM65	9.13	0.859	89	100
TMEM65	8.13	0.8633	91	98
TMEM65	6.85	0.8345	-1120	-1113
TMEM65	6.74	0.8086	-1122	-1111
TMEM65	5.78	0.8102	-742	-735

C

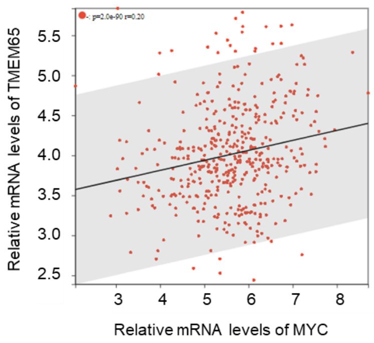

D

TMEM65 promoter (-2000 to +200 bp)

R1	R2	R3
-1230 to -1069	-837 to -659	-33 to 124

Supplementary Fig. S4. MYC is a potential upstream transcription factor to transactivate TMEM65 in TNBC cells

(**A**) The predicted putative binding motif of transcription factor MYC on TMEM65 promoter using transcription factor binding site database JASPAR (https://jaspar.elixir.no/).

(**B**) The predicted score for MYC binding to TMEM65 promoter.

(**C**) The association of the expression levels of TMEM65 with MYC in FUSCC-TNBC RNA-Seq dataset.

(**D**) Diagrammatic sketch showing the regions of TMEM65 promoter that were cloned for luciferase assays.

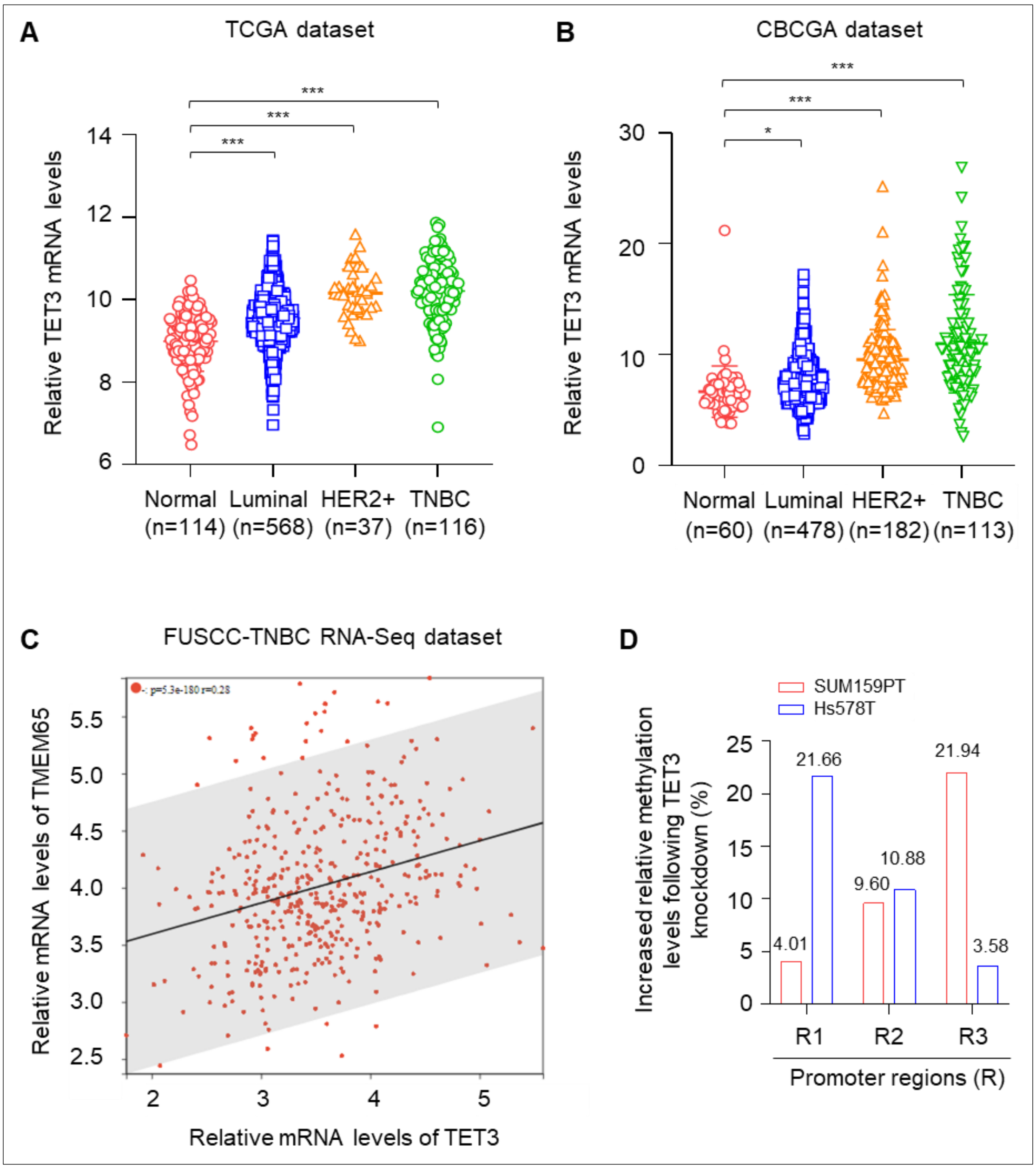

Supplementary Fig. S5. TET3 regulates TMEM65 expression in TNBC cells

(**A and B**) The expression levels of TET3 in normal tissues and breast cancer in the TCGA (A) and CBCGA (B) datasets.

(**C**) The association of the expression levels of TMEM65 with TET3 in FUSCC-TNBC RNA-Seq dataset.

(**D**) Pyrosequencing analysis of the increased relative methylation levels of TMEM65 promoter following knockdown of TET3 at the three tested regions (R) in SUM159PT and Hs578T cells.

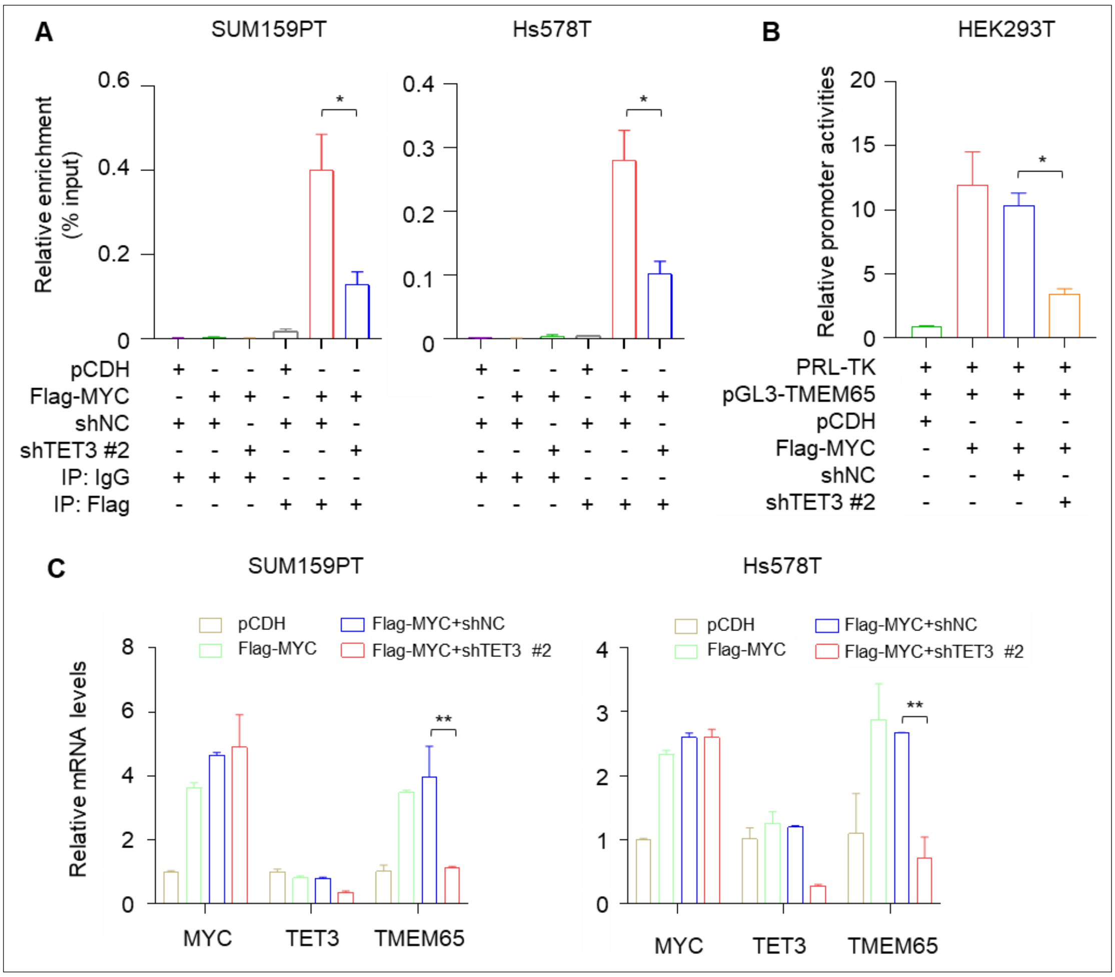

Supplementary Fig. S6. MYC and TET3 cooperate to activate TMEM65 expression in TNBC cells

(**A**) ChIP-qPCR assays were conducted to examine the effects of TET3 knockdown on the recruitment of MYC to TMEM65 promoter.

(**B**) Luciferase assays were carried out to determine the effects of knockdown of TET3 on MYC-activated promoter activities of TMEM65.

(**C**) Detection of the effects of TET3 knockdown on MYC-mediated upregulation of TMEM65 mRNA levels by RT-qPCR assays.

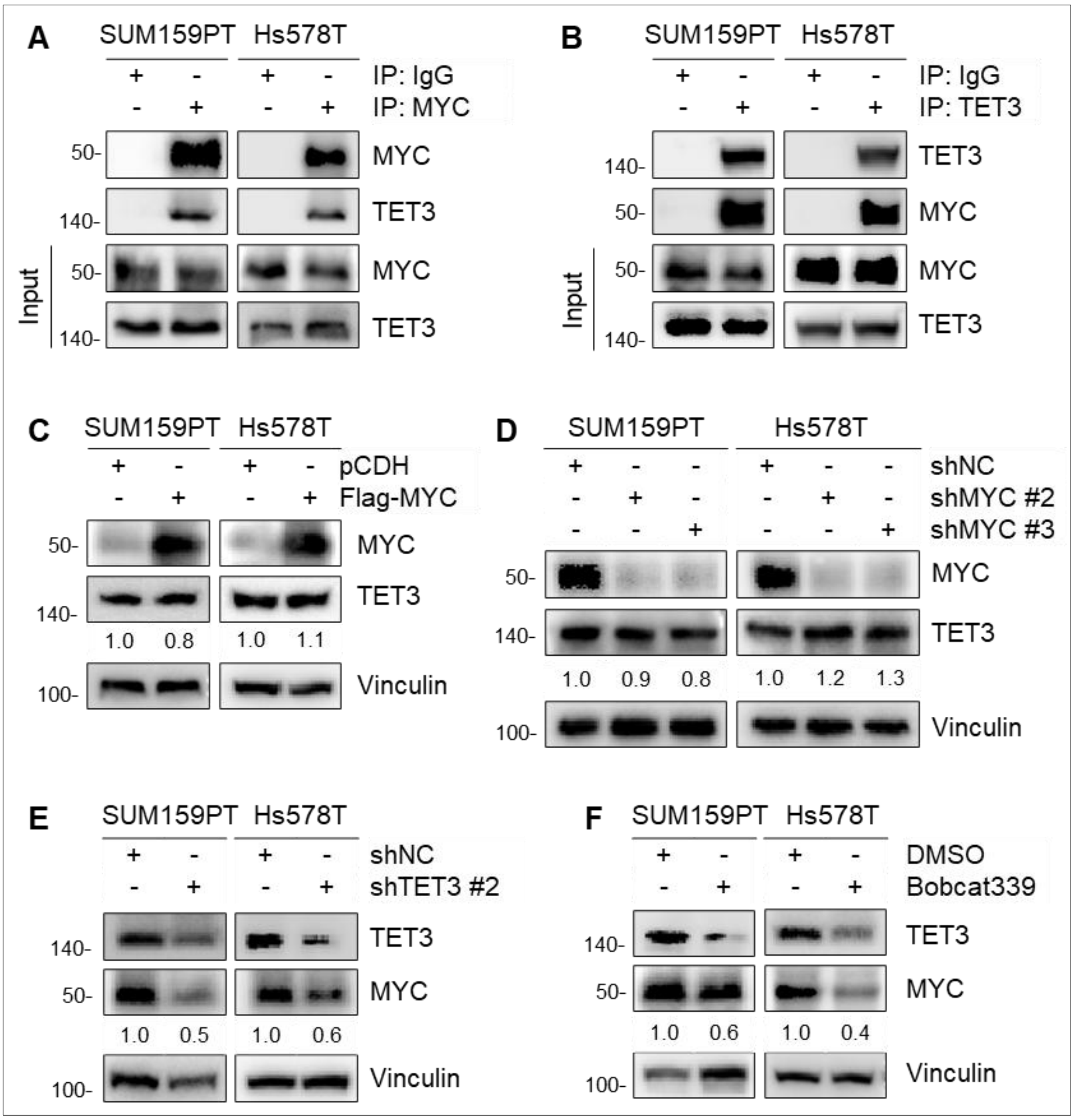

Supplementary Fig. S7. The potential functional interaction between TET3 and MYC in TNBC cells

(**A and B**) Reciprocal IP assays were conducted using an anti-MYC (A) or anti-TET3 (B) antibody to examine the interaction between MYC and TET3 at the endogenous level.

(**C and D**) Detection of the effects of overexpression (C) or knockdown (D) of MYC on TET3 expression levels by immunoblotting assays.

(**E and F**) Detection of the effects of knockdown (E) or pharmacological inhibition (F) of TET3 on the protein expression levels of MYC by immunoblotting assays.

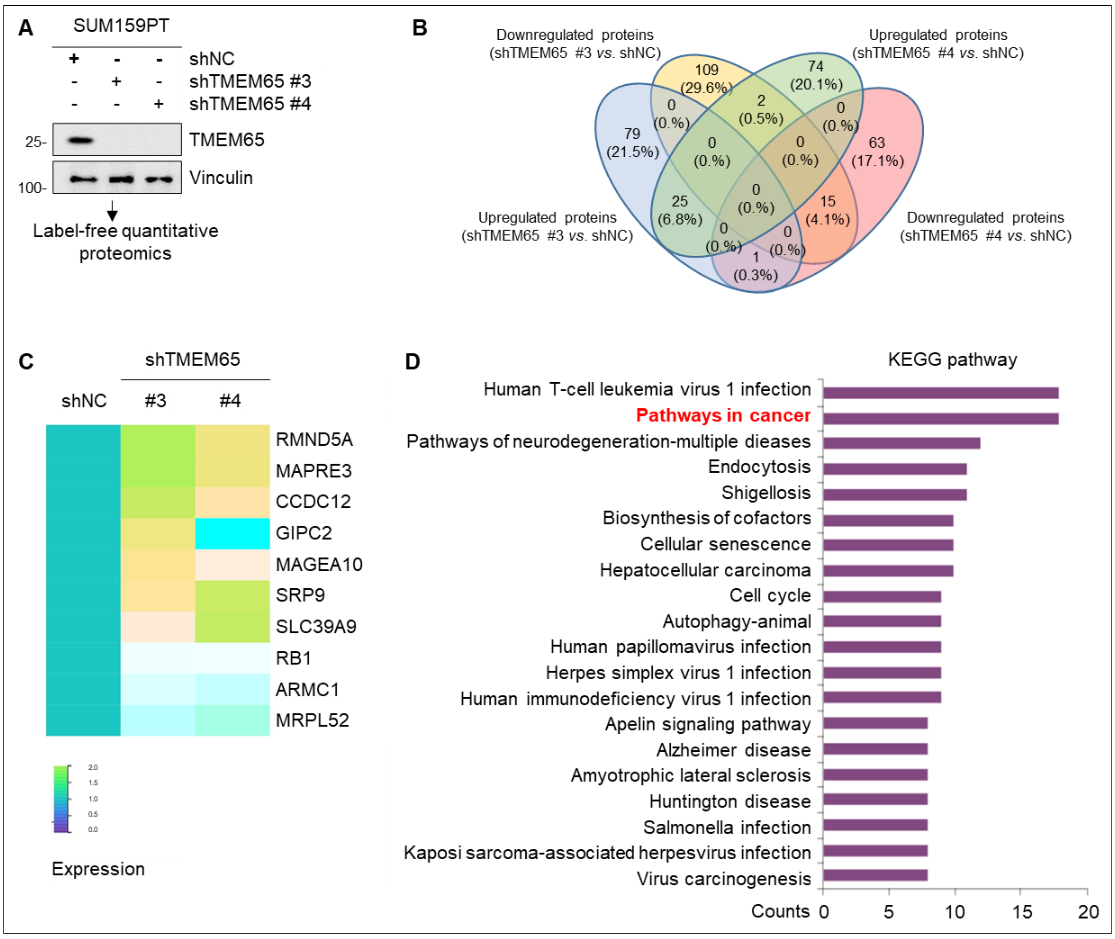

Supplementary Fig. S8. Identification of downstream targets of TMEM65 by quantitative proteomic assays

(**A**) Immunoblotting assays were performed to validate the expression status of TMEM65 in SUM159T cells stably expressing shNC and shTMEM65. The validated cells were subjected to label-free quantitative proteomic assays.

(**B**) Venny diagram showing the number of the differentially expressed proteins after knockdown of TMEM65.

(**C**) The top 10 up-regulated proteins following knockdown of TMEM65.

(**D**) KEGG pathway analysis of the differentially expressed proteins after knockdown of TMEM65.

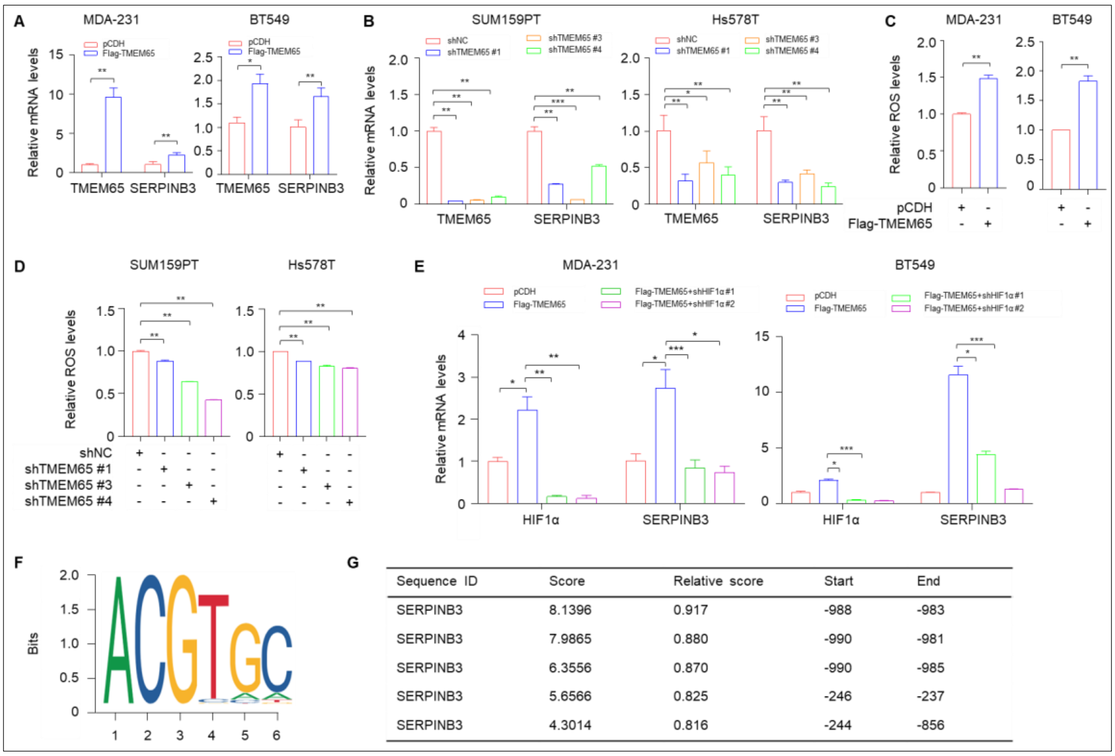

Sequence ID	Score	Relative score	Start	End
SERPINB3	8.1396	0.917	-988	-983
SERPINB3	7.9865	0.880	-990	-981
SERPINB3	6.3556	0.870	-990	-985
SERPINB3	5.6566	0.825	-246	-237
SERPINB3	4.3014	0.816	-244	-856

Supplementary Fig. S9. TMEM65 transactivates oncogene SERPINB3 through activating OXPHOS-ROS-HIF1α pathway

(**A-B**) TNBC cells with ectopic expression (A) or knockdown (B) of TMEM65 were subjected to RT-qPCR assays to detect the mRNA levels of SERPINB3.

(**C-D**) ROS levels were determined in TNBC cells with ectopic expression (C) or knockdown (D) of TMEM65.

(**E**) MDA-231 and BT549 cells stably expressing pCDH, Flag-TMEM65, shHIF1α alone or in combination were subjected to RT-qPCR assays to detect the mRNA levels of SERPINB3.

(**F**) The predicted putative binding motif of transcription factor HIF1α on SERPINB3 promoter using transcription factor binding site database JASPAR (https://jaspar.elixir.no/).

(**G**) The predicted score for HIF1α binding onto SERPINB3 promoter.

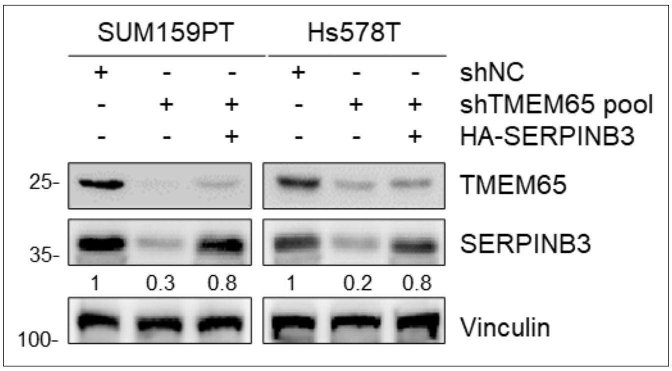

Supplementary Fig. S10. Establishment of stable cell lines with re-expression of HA-SERPINB3 in TMEM65-depleted TNBC cells

Immunoblotting assays were performed to validate the expression status of TMEM65 and SERPINB3 in SUM159PT and Hs578T cells stably expressing shNC, shTMEM65, HA-SERPINB3 alone or combination.

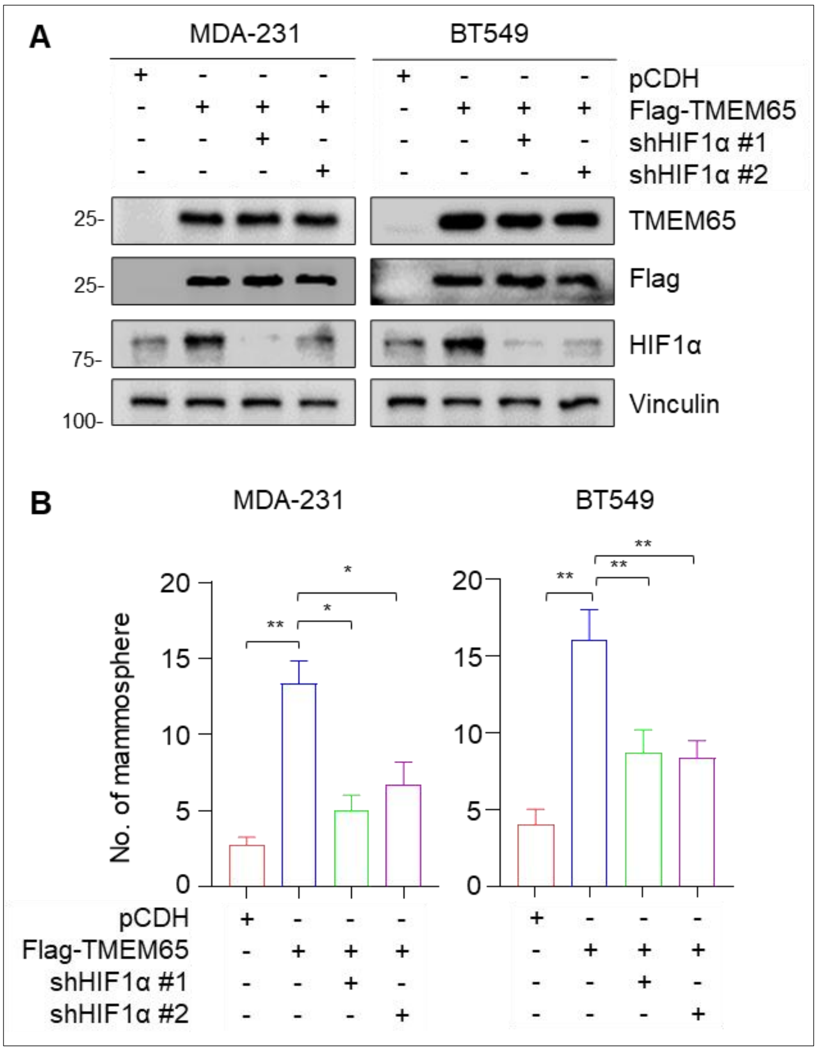

Supplementary Fig. S11. Knockdown of HIF1α attenuates TMEM65-induced the sphere formation ability.

(**A-B**) MDA-231 and BT549 cells stably expressing pCDH, Flag-TMEM65, shHIF1α alone or in combination (A) were subjected to mammosphere assays to detect the sphere formation ability (B).

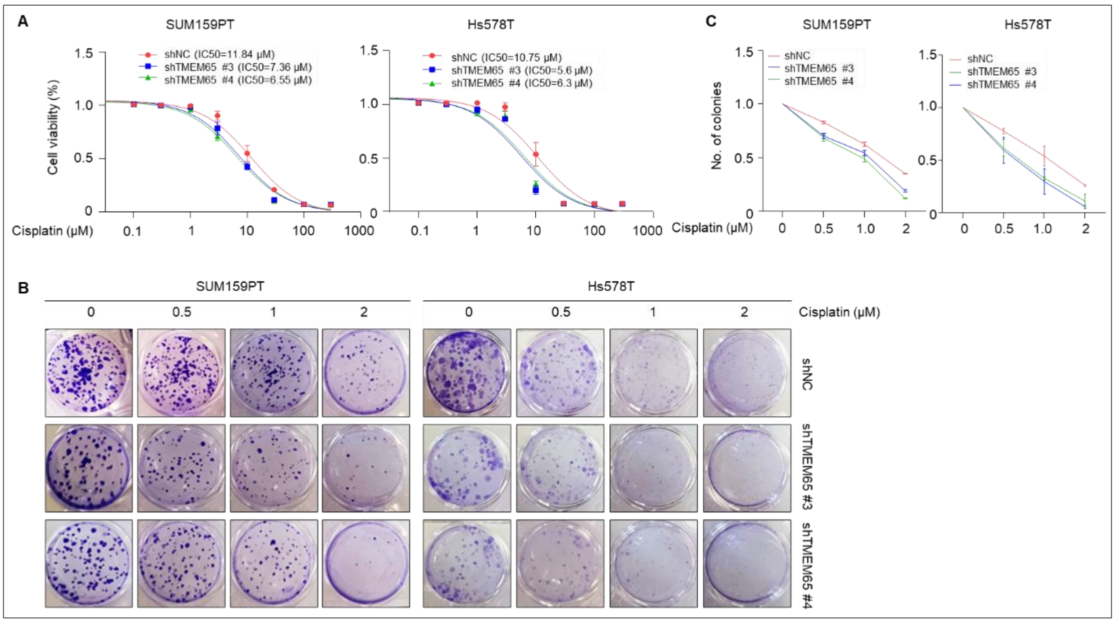

Supplementary Fig. S12. TMEM65 promote resistance of TNBC cells to cisplatin *in vitro*

(**A-C**) SUM159T and Hs578T cells stably expressing shNC and shTMEM65 were treated with or without increasing doses of cisplatin and subjected to CCK-8 (A) and colony formation (B-C) assays. The representative images of survival colonies and corresponding quantitative results are shown in B and C, respectively.

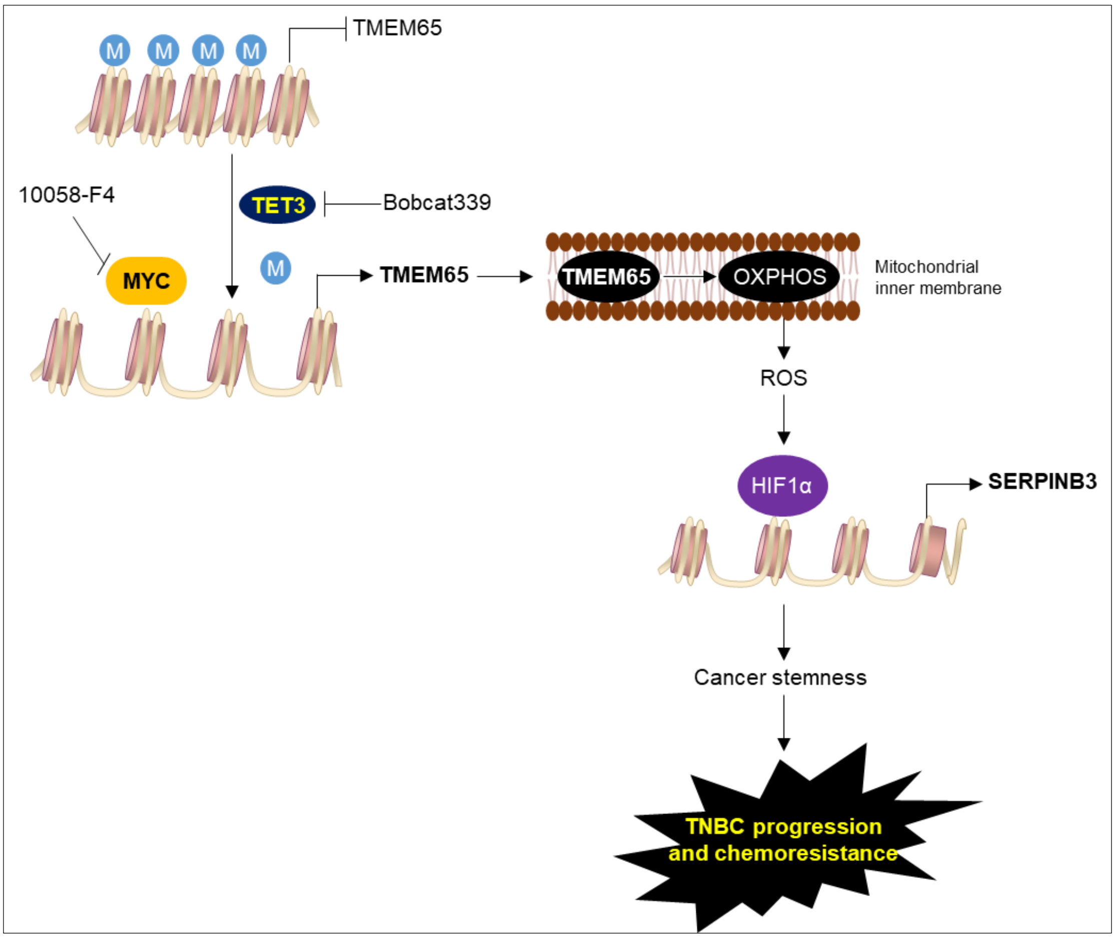

Supplementary Fig. S13. The proposed working model.

Transcription factor MYC and DNA demethylase TET3 coordinately upregulate TMEM65 in TNBC. Upregulated TMEM65 acts as a novel regulator of TNBC stemness by activating OXPHOS-SERPINB3 pathway to promote TNBC progression and chemoresistance. Schematic diagram of chromatin structure was created using the ScienceSlides program. M, methylation.

Supplementary Tables

Supplementary Table S1. Chemical reagents used in this study

**Chemical reagents**	**Vendors**	**Cat#**
10058-F4	Selleck	S7153
Bobcat339	Selleck	S6682
N-acetylcysteine (NAC)	MCE	HY-B0215
Cisplatin	Selleck	S1166

Supplementary Table S2. The primers used for molecular cloning of expression vectors

Genes	Primers	Sequences
Flag-TMEM65	Forward	ACCTCCATAGAAGATTCTAGAGCCACCATGTCCCGGCTGCTGC
	Reverse	ATCCGATTTAAATTCGAATTCTTACTTGTCATCGTCGTCCTTGTAATCACTTTTCGTTTCCAGTTTTTCATCTTCTTCAC
Flag-HIF1α	Forward	ACCTCCATAGAAGATTCTAGAGCCACCTGGAGGGCGCCGGC
	Reverse	ATCCGATTTAAATTCGAATTCTTACTTGTCATCGTCGTCCTTGTAATCAACTTGATCCAAAGCTCTGAGTAATTCTTCA
Flag-MYC	Forward	ACCTCCATAGAAGATTCTAGAGCCACCATGCCCCTCAACGTTAGCTTC
	Reverse	ATCCGATTTAAATTCGAATTCTTACTTGTCATCGTCGTCCTTGTAATCCGCACAAGAGTTCCGTAGCTG
HA-SERPINB3	Forward	GGATCTATTTCCGGTGAATTCGCCACCATGAATTCACTCAGTGAAGCCAACAC
	Reverse	GGGATCCGCGGCCGCTCTAGATTAAGCGTAGTCTGGGACGTCGTATGGGTACGGGGATGAGAATCTGCCATAGA

Supplementary Table S3. shRNA sequences targeting TMEM65, TET3, MYC, and HIF1α

Genes	Primers	Sequences
shTMEM65 #1	Forward	CCGGGCCTGTCAATTCCTGATCTCACTCGAGTG AGATCAGGAATTGACAGGCTTTTTG
	Reverse	AATTCAAAAAGCCTGTCAATTCCTGATCTCACT CGAGTGAGATCAGGAATTGACAGGC
shTMEM65 #3	Forward	CCGGGTATTCATCCACAATGCGATACTCGAGTAT CGCATTGTGGATGAATAC TTTTTG
	Reverse	AATTCAAAAAGTATTCATCCACAATGCGATACTC GAGTATCGCATTGTGGATGAATAC
shTMEM65 #4	Forward	CCGGCACAATGCGATACCTTTCATACTCGAGTAT GAAAGGTATCGCATTGTGTTTTTG
	Reverse	AATTCAAAAACACAATGCGATACCTTTCATACTC GAGTATGAAAGGTATCGCATTGTG
shTET3 #2	Forward	CCGGGAAAGATGAAGGTCCATATTACTCGAG TAATATGGACCTTCATCTTTCTTTTTG
	Reverse	AATTCAAAAAGAAAGATGAAGGTCCATATTACTC GAGTAATATGGACCTTCATCTTTC
shMYC #1	Forward	CCGGCCTGTGCCACTAAACTACATTCTCGAGAAT GTAGTTTAGTGGCACAGG TTTTTG
	Reverse	AATTCAAAAACCTGTGCCACTAAACTACATTCTC GAGAATGTAGTTTAGTGGCACAGG
shMYC #2	Forward	CCGGCGAGGACATCTGGAAGAAATTCTCGAG AATTTCTTCCAGATGTCCTCGTTTTTG
	Reverse	AATTCAAAAACGAGGACATCTGGAAGAAATTCT CGAGAATTTCTTCCAGATGTCCTCG
shHIF1α #1	Forward	CCGGCCGCTGGAGACACAATCATATCTCGAG ATATGATTGTGTCTCCAGCGGTTTTTG
	Reverse	AATTCAAAAACCGCTGGAGACACAATCATATCTC GAGATATGATTGTGTCTCCAGCGG
shHIF1α #2	Forward	CCGGCCAGTTATGATTGTGAAGTTACTCGAG TAACTTCACAATCATAACTGGTTTTTG
	Reverse	AATTCAAAAACCAGTTATGATTGTGAAGTTACTC GAGTAACTTCACAATCATAACTGG

Supplementary Table S4. The primers used for RT-qPCR

Genes	Primers	Sequences
TMEM65	Forward	GGGCACACACCCCAAGAA G
	Reverse	ACCCTACGAAAGGTATCGCATG
SERPINB3	Forward	CGCGGTCTCGTGCTATCTG
	Reverse	ATCCGAATCCTACTACAGCGG
TET3	Forward	TGCGATTGCGTCGAACA
	Reverse	TGCGGATCACCCACTTTG
HIF1α	Forward	GAACGTCGAAAAGAAAAGTCTCG
	Reverse	CCTTATCAAGATGCGAACTCACA

Supplementary Table S5. Antibodies used in this study

Antibodies	Vendors	Cat#	Host
TMEM65	Proteintech	21913-1-AP	Rabbit
MYC	Proteintech	10828-1-AP	Rabbit
SERPINB3	Proteintech	11428-1-AP	Rabbit
HIF1α	Abclonal	A11945	Rabbit
TET3	Abclonal	GT859	Mouse
ALDH1A1	Proteintech	60171-1-Ig	Mouse
CD44	Proteintech	60224-1-Ig	Mouse
Nanog	Proteintech	67255-1-Ig	Mouse
HA	CST	3724S	Rabbit
Flag	Sigma-Aldrich	F1804	Mouse
Vinculin	Sigma-Aldrich	V9131	Mouse

Supplementary Table S6. Primers used for cloning TMEM65 and SERPINB3 promoter

Genes	Primers	Sequences
pGL3-TMEM65-F1	Forward	CGAGCTCTTACGCGTGCTAGCCTGGAGGTGGTTGGGAATGG
pGL3-TMEM65-R1	Reverse	ACTTAGATCGCAGATCTCGAGAACTGAATAATGGAGGATTGTTACCACTAA
pGL3-TMEM65-F2	Forward	CGAGCTCTTACGCGTGCTAGCGTTTCCGGGCTGAGTGCAGCACTGTTTATG
pGL3-TMEM65-R2	Reverse	ACTTAGATCGCAGATCTCGAGTCGCCAAGTCAGCGCCCACCG
pGL3-TMEM65-F3	Forward	CGAGCTCTTACGCGTGCTAGCAGGGCGCAGCTGGGGCG
pGL3-TMEM65-R3	Reverse	ACTTAGATCGCAGATCTCGAGTGTGTCTCTGTCAGTCTGATCTTCCAGAGGATGA
pGL3-SERPINB3-F	Forward	CGAGCTCTTACGCGTGCTAGCCTCTTCTCAGTGTCAGCCTGTTAA
pGL3-SERPINB3-R	Reverse	ACTTAGATCGCAGATCTCGAGTTCTCAAAAATAATTCATGTTATTAAGCTGGAGA

Supplementary Table S7. Primers used for ChIP-PCR assays

Genes	Primers	Sequences
TMEM65-F1	Forward	CAGAGGCATCACCTTGGAAT
TMEM65-R1	Reverse	CCCACTTCACACCTCCTGAA
TMEM65-F2	Forward	TAAGGGTGCCTAAGCGTCAT
TMEM65-R2	Reverse	CTCTGTGCTAAAGCGGGAAG
TMEM65-F3	Forward	GCACCTCCTTCCCCTTTC
TMEM65-R3	Reverse	GCCAGCTTTCTCACCCTCTA
SERPINB3-F	Forward	TCAGTGTCAGCCTGTTAACTCAA
SERPINB3-R	Reverse	TTGCAGCCACCATGTAAGAA

Supplementary Table S8. The PCR and sequencing primers used for pyrosequencing

Primers	Sequences
TMEM65 Pyro-F-1	TTTTTGGGTAGGGGTGGAAT
TMEM65 Pyro-R-1	CCCCAACCTAAAACCACAT
TMEM65 Pyro-Seq-1	GGTAGGGGTGGAATT
TMEM65 Pyro-F-2	TTTTTGGAAGATTAGATTGATAGAGATA
TMEM65 Pyro-R-2	CCCCTCCCATAAAAAAAACTTTCTCTAA
TMEM65 Pyro-Seq-2	AGATTGATAGAGATATATATTAG
TMEM65 Pyro-F-3	GGGTTTTTTGGAAAGGAAGTG
TMEM65 Pyro-R-3	CCCCCAAATCCTCCTACCAAAC
TMEM65 Pyro-Seq-3	GAAAGGAAGTGGGGA
